# Interactions between Cognitive, Affective, and Respiratory Profiles in Chronic Respiratory Disorders: A Cluster Analysis Approach

**DOI:** 10.3390/diagnostics14111153

**Published:** 2024-05-30

**Authors:** Iulian-Laurențiu Buican, Victor Gheorman, Ion Udriştoiu, Mădălina Olteanu, Dumitru Rădulescu, Dan Marian Calafeteanu, Alexandra Floriana Nemeş, Cristina Călăraşu, Patricia-Mihaela Rădulescu, Costin-Teodor Streba

**Affiliations:** 1 U.M.F. Doctoral School Craiova, University of Medicine and Pharmacy of Craiova, 200349 Craiova, Romania; laurentiu.buican@umfcv.ro; 2 Leamna Pulmonology Hospital, 207129 Leamna, Romania; cristina.calarasu@umfcv.ro (C.C.); paty_miha@yahoo.com (P.-M.R.); costin.streba@umfcv.ro (C.-T.S.); 3 Department of Psychiatry, University of Medicine and Pharmacy of Craiova, 200349 Craiova, Romania; victor.gheorman@umfcv.ro (V.G.); ion.udristoiu@umfcv.ro (I.U.); 4 Department of Orthodontics, University of Medicine and Pharmacy of Craiova, 200349 Craiova, Romania; madalina.olteanu@umfcv.ro; 5 Department of Surgery, The Military Emergency Clinical Hospital ‘Dr. Stefan Odobleja’ Craiova, 200749 Craiova, Romania; 6 Department of Neonatology, ‘Louis Ţurcanu’ Clinical Emergency Hospital for Children, 300011 Timişoara, Romania; nemesalexandrafloriana@gmail.com; 7 Department of Pulmonology, University of Medicine and Pharmacy of Craiova, 200349 Craiova, Romania

**Keywords:** chronic obstructive pulmonary disease, affective disorders, asthma, cognitive disorders

## Abstract

This study conducted at Leamna Pulmonology Hospital investigated the interrelations among cognitive, affective, and respiratory variables within a cohort of 100 patients diagnosed with chronic respiratory conditions, utilizing sophisticated machine learning-based clustering techniques. Spanning from October 2022 to February 2023, hospitalized individuals confirmed to have asthma or COPD underwent extensive evaluations using standardized instruments such as the mMRC scale, the CAT test, and spirometry. Complementary cognitive and affective assessments were performed employing the MMSE, MoCA, and the Hamilton Anxiety and Depression Scale, furnishing a holistic view of patient health statuses. The analysis delineated three distinct clusters: Moderate Cognitive Respiratory, Severe Cognitive Respiratory, and Stable Cognitive Respiratory, each characterized by unique profiles that underscore the necessity for tailored therapeutic strategies. These clusters exhibited significant correlations between the severity of respiratory symptoms and their effects on cognitive and affective conditions. The results highlight the benefits of an integrated treatment approach for COPD and asthma, which is personalized based on the intricate patterns identified through clustering. Such a strategy promises to enhance the management of these diseases, potentially elevating the quality of life and everyday functionality of the patients. These findings advocate for treatment customization according to the specific interplays among cognitive, affective, and respiratory dimensions, presenting substantial prospects for clinical advancement and pioneering new avenues for research in the domain of chronic respiratory disease management.

## 1. Introduction

### 1.1. General Context

In recent decades, the global public health challenge posed by the rising prevalence of chronic respiratory diseases, including asthma and chronic obstructive pulmonary disease (COPD), has become increasingly prominent. Notably, studies indicate that in 2015, approximately 3.2 million individuals succumbed to COPD worldwide—an 11.6% increase since 1990. Despite this, age-standardized mortality rates saw a 41.9% decline, reflecting advancements in treatment options and a heightened awareness of risk factors. However, during the same period, the absolute prevalence of COPD surged by 44.2%, even as the age-standardized prevalence decreased by 14.7% [1].

By 2017, an estimated 544.9 million people globally were afflicted with chronic respiratory diseases, marking a 39.8% increase from 1990. This prevalence varies significantly by region, with high-income areas being the most affected and regions like sub-Saharan Africa and South Asia the least. Smoking remains a critical risk factor, accountable for approximately 73.3% of the total disability-adjusted life years (DALYs) attributable to COPD, underscoring the critical need for robust prevention and control measures [2].

The demographic shift towards an aging population also plays a crucial role in the increasing prevalence of respiratory diseases, particularly in affluent regions. This demographic trend necessitates tailored management strategies and sufficient resources to address the rising disease incidence, highlighting the importance of innovative approaches in medical research and public health policy [1].

Economically, respiratory diseases, including COPD, impose a significant burden, evidenced by direct treatment costs and indirect losses such as reduced productivity and diminished quality of life. Regional studies illustrate this impact vividly: for instance, in Korea, lost productivity, including presenteeism, incurs costs of approximately USD 8853 per patient annually [3]. Similarly, in urban areas of China, direct medical costs are estimated at CNY 11,744 (around USD 1660) per year, with additional non-medical costs of approximately CNY 1570 (around USD 222) [4].

In Southeast Europe, countries like Serbia and Bulgaria report a substantial economic strain due to COPD, with indirect costs from productivity loss and early retirement dominating, reflecting broader economic challenges such as lower average incomes and high unemployment rates among youth [5]. In Bulgaria, the impact of COPD is particularly pronounced, with about 69% of patients being male and 40% having occupational risk factors, where indirect costs are three times that of direct costs. This disparity highlights the urgency of implementing effective prevention and management strategies to alleviate economic pressures and enhance patient quality of life [6].

Moreover, the management of respiratory diseases like COPD is complicated by frequent comorbidities, including cardiovascular diseases and diabetes, which not only heighten the risk of acute exacerbations and mortality but also complicate clinical management. Patients with COPD, for instance, have up to a third higher risk of developing cardiovascular comorbidities, hypertension, and diabetes, necessitating an integrated approach to treatment [7].

COPD is also associated with a variety of other conditions such as pulmonary artery disease and malnutrition, which directly result from lung pathology. Other comorbid conditions like systemic venous thromboembolism, anxiety, depression, osteoporosis, obesity, metabolic syndrome, sleep disorders, and anemia, although not directly linked to COPD from a pathophysiological perspective, frequently occur due to chronic systemic inflammation typical of these conditions [8]. This comorbidity burden exacerbates COPD-related morbidity, leading to frequent hospitalizations and substantial healthcare costs, thus complicating patient management overall. These complexities underscore the need for personalized, multidisciplinary treatment strategies in the care of patients with chronic respiratory diseases.

Research by Martinez et al. demonstrates that COPD is not only associated with preexisting disabilities but also with the onset of new disability cases, with cognitive disorders intensifying these associations. Data from the Health and Retirement Study revealed that patients with COPD show a higher prevalence and incidence of disability compared to those without the condition, with impacts comparable to or greater than those seen in other major chronic diseases like stroke or diabetes [9].

In a related study, Salik et al. explored the impact of cognitive function on the quality of life among COPD patients, finding that while cognitive function remains relatively unaffected in those with mild hypoxemia, their quality of life is significantly reduced compared to healthy individuals. This highlights the profound impact of COPD on quality of life and underscores the necessity of addressing cognitive and affective consequences within disease management frameworks. These studies advocate for an integrated research and treatment approach that includes the evaluation and management of cognitive and affective outcomes to optimize clinical results and improve patient quality of life [10].

### 1.2. The Relevance of the Intersection between Psychiatry and Pulmonology

To underscore the significance of the intersection between psychiatry and pulmonology, it is crucial to recognize the heightened prevalence of psychiatric disorders among patients with chronic respiratory conditions. European studies, for instance, have consistently found that disorders such as anxiety and depression are prevalent among individuals with chronic respiratory diseases, often complicating both the management and prognosis of these conditions. A notable study conducted across various hospitals in the United Kingdom demonstrated that as many as 74.6% of patients with advanced respiratory diseases had documented comorbid psychiatric conditions prior to receiving care from the Advanced Lung Disease Service [11]. This finding accentuates the necessity for early and personalized interventions aimed at enhancing both disease management and the quality of life for these patients, reflecting the imperative for an integrated treatment approach that considers both the physical and psychological dimensions of chronic respiratory diseases.

A cross-sectional study from general hospitals in Jiangsu Province, China, further emphasized the high prevalence of depression (46%) and anxiety (25.34%) among patients with chronic respiratory diseases. Identified risk factors included low body mass index, sleep disorders, limitations in physical activity, and adverse life events, highlighting the intricate interplay between physical health and mental well-being [12]. This study reinforces the deep-seated connection between chronic physical health conditions and mental health, suggesting that the management of respiratory diseases should encompass psychological interventions.

Additional research conducted in public sector hospitals revealed an alarmingly high prevalence of moderate to severe mental disorders, including depression, among patients with COPD. These findings underscore the critical importance of incorporating psychological counseling services within health promotion programs to mitigate the impact of depression and enhance life quality for these patients [13]. Therefore, adopting an integrated approach in treating respiratory diseases, which recognizes and addresses the complex mental health needs associated with these conditions, is essential for developing effective strategies that alleviate both the physical and psychological symptoms of chronic respiratory diseases.

### 1.3. Study Objectives

Our research aims to delve into the complexity of the interactions between cognitive function, affective state, and the severity of respiratory symptoms in patients with chronic respiratory diseases. Through detailed cluster analysis, this study intends to categorize patients into homogeneous groups, reflecting key differences based on a complex set of characteristics. This methodology will not only aid in uncovering hidden patterns and correlations within the data but will also enhance our understanding of how these variables interact and affect disease progression and treatment responses.

A central element of this study involves evaluating the associations between characteristics identified within the clusters. We aim to establish the statistical relationships between cognitive, affective, and respiratory disease severity variables to identify predictive factors that may influence patient management. This information is pivotal for personalizing therapeutic approaches, ensuring that interventions address multiple facets of the disease simultaneously.

Furthermore, our research will explore the clinical implications of the findings obtained through clustering. We plan to analyze how these insights could influence medical practice, suggesting modifications to intervention strategies based on the specific characteristics of each patient group. This effort will contribute to optimizing treatment protocols and improving the quality of life for patients, highlighting the importance of an integrated approach in the management of chronic respiratory diseases.

Additionally, this study aims to develop predictive models based on cluster analysis, which will aid in anticipating disease progression and validate the reliability of measurement tools used specifically in the context of respiratory diseases. These models will equip clinicians with essential tools for making rapid, well-informed decisions, enhancing clinical interventions, and ensuring a more effective and tailored approach to meet patient needs (Table 1).

## 2. Materials and Methods

This prospective pilot study was initiated to explore the complex interrelations among cognitive, affective, and respiratory profiles in patients with chronic respiratory disorders. The research was conducted at Leamna Pulmonology Hospital, a premier center for the treatment of respiratory conditions in Romania, during the period from October 2022 to February 2023. Hospitalized patients with a confirmed diagnosis of asthma or COPD were evaluated under the strict supervision of medical staff and researchers.

The objective of the study was to delineate the patterns of interaction among cognitive, affective, and respiratory variables and to ascertain their contributions to the progression and management of chronic respiratory diseases. As a pilot study, it aimed to validate preliminary hypotheses and lay the groundwork for more comprehensive future research that could expand and refine these initial findings.

Ethical approval for the study was granted by the Ethics Committee of the University of Medicine and Pharmacy in Craiova and Leamna Pulmonology Hospital, adhering to all applicable national and international ethical standards. The approval numbers were 196/17 February 2022 and 6189/13 October 2022.

### 2.1. Inclusion and Exclusion Criteria

The inclusion and exclusion criteria were meticulously defined to ensure the coherence and accuracy of data in this prospective pilot study. Eligible participants were required to have a confirmed diagnosis of asthma or chronic obstructive pulmonary disease (COPD) and to be able to provide written informed consent, confirming their comprehensive understanding of the study’s purpose, procedures, and potential risks. Participants were required to be adults over 18 years of age and capable of adhering to the study protocols during their hospitalization.

**Figure 1:**
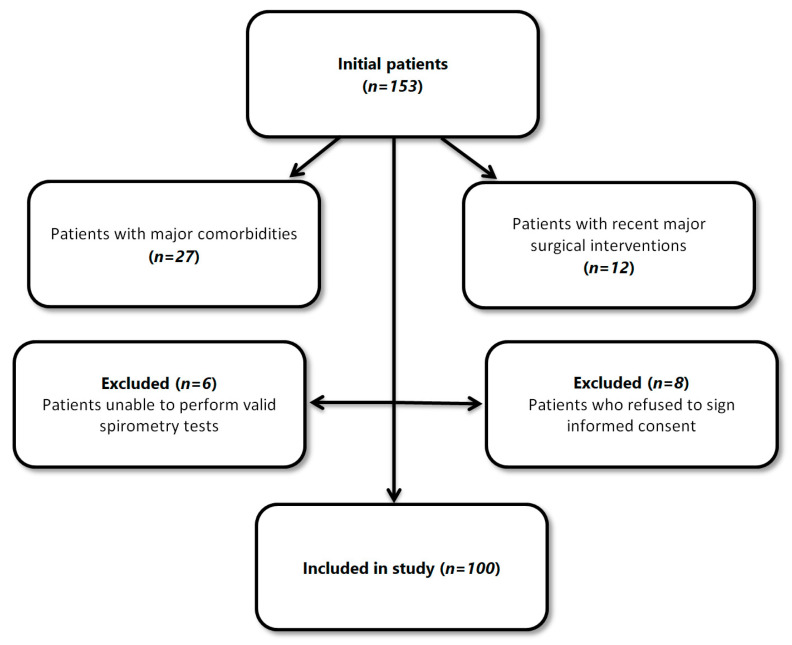
Flow chart of patient inclusion.

Initially, 153 patients were identified as potential participants for the study. After applying our stringent exclusion criteria, the cohort was narrowed down to 100 patients suitable for detailed analysis. Exclusions were made for various reasons: 27 patients were excluded due to major comorbidities, 12 due to recent major surgical interventions, 6 were unable to perform valid spirometry tests, a critical component of our respiratory assessments, and 8 declined to sign the informed consent (Figure 1).

Patients with severe comorbidities that could confound data interpretation, such as significant cardiac conditions, active cancer, or uncontrolled major psychiatric disorders, were excluded. Additionally, those who had undergone major surgical interventions within the past six months or who refused to sign informed consent were also excluded.

These criteria ensured that the data collected were representative and valid for subsequent analysis, allowing for the precise interpretation of the interactions among the cognitive, affective, and respiratory profiles of the participants.

We included a total of 100 patients in the study after applying the inclusion and exclusion criteria.

### 2.2. Respiratory Assessment

Respiratory assessment was a critical component of this study, providing essential data on lung function in patients with chronic respiratory disorders. This included the use of standardized tools and techniques to measure and analyze various aspects of respiratory function.

Modified Medical Research Council (mMRC) Dyspnea Scale

The Modified Medical Research Council (mMRC) Dyspnea Scale is a tool used to assess the severity of dyspnea (difficulty breathing) in patients with chronic respiratory conditions. The mMRC scale ranges from 0 to 4, where 0 indicates dyspnea only with strenuous exercise, and 4 represents severe dyspnea that prevents the patient from leaving the house or performing any activity without major difficulty. Specifically, a score of 1 on the mMRC scale indicates dyspnea when climbing a slope or hill; a score of 2 indicates the need to stop for breath when walking on level ground or that the patient walks slower than people of the same age due to dyspnea; a score of 3 indicates stopping for breath after walking about 100 m or a few minutes on level ground; and a score of 4 indicates dyspnea so severe that it prevents the patient from leaving the house or causes significant difficulty in performing any activity [14].

COPD Assessment Test (CAT)

The COPD Assessment Test (CAT) is a questionnaire designed to measure the impact of chronic obstructive pulmonary disease (COPD) symptoms on patients’ daily activities. The CAT consists of eight questions, each with a scale from 0 to 5, resulting in a total score ranging from 0 to 40. The questions cover various aspects of the disease, including cough, sputum production, chest tightness, difficulty climbing stairs, daily activities, confidence in leaving the house due to the condition, sleep, and energy levels. Scores are classified from a low level (below 10), indicating mild symptoms with minimal impact on daily life, to a very high level (above 30), indicating very severe symptoms that severely affect daily life [15].

Spirometry and Forced Expiratory Volume in the First Second (FEV1)

Spirometry is a pulmonary function test that measures the amount of air a person can exhale and is used to diagnose and monitor respiratory conditions such as asthma and COPD. A key indicator measured during spirometry is the forced expiratory volume in the first second (FEV1), which represents the volume of air a person can forcibly exhale in the first second after a full inhalation. FEV1 is used to classify the severity of airway obstruction, from mild obstruction (FEV1 above 70% of the predicted value) to very severe obstruction (FEV1 below 35% of the predicted value). Spirometry is performed using a device called a spirometer, such as the Spirolab IV, which records air volumes and flow rates, providing precise data on lung function [16].

Use of These Tools in the Study

These tools were used to provide a comprehensive and multidimensional assessment of each participant’s respiratory status. The mMRC scale was used to assess the severity of dyspnea, the CAT test measured the impact of symptoms on daily activities, and spirometry, by measuring FEV1, provided crucial data for diagnosing and monitoring asthma and COPD. These assessments provided valuable data for further analysis, contributing to the understanding of interactions between the respiratory function, cognitive state, and affective state of the patients.

### 2.3. Psychiatric Evaluation

The psychiatric assessment was essential for understanding the complex interactions between the mental state and respiratory conditions of the patients. We used a combination of validated tools to ensure a thorough and multidimensional evaluation of cognitive and affective functions.

Mini-Mental State Examination (MMSE)

The Mini-Mental State Examination (MMSE) is a widely used screening test in clinical practice for the initial assessment of cognitive function. This test measures various cognitive components, including orientation in time and space, immediate memory, attention and calculation, object recognition, and language. MMSE scores are interpreted to determine the degree of cognitive impairment, divided into four categories: normal (30–25), mild (24–20), moderate (19–10), and severe (below 10) [17].

Montreal Cognitive Assessment (MoCA)

The Montreal Cognitive Assessment (MoCA) was used as a complementary tool to the MMSE, aimed at detecting subtle cognitive dysfunctions that are not always evident in the MMSE. MoCA evaluates more complex cognitive domains, including focused and distributed attention, executive functions, short-term memory, language, visuospatial construction skills, and temporal and spatial orientation. The test is scored on a scale of 0 to 30, with results divided into four severity categories: normal (30–26), mild (25–18), moderate (17–10), and severe (below 10) [18].

Hamilton Anxiety and Depression Scale (HADS)

The Hamilton Anxiety and Depression Scale (HADS) was used to evaluate affective components such as anxiety and depression. HADS is useful in distinguishing the severity levels of these disorders, with separate sections dedicated to measuring anxiety and depression. HADS questions focus on somatic and psychological anxiety symptoms, feelings of guilt, suicidal thoughts, insomnia, and overall daily functioning. Scores are grouped into categories from normal (0–7) to severe (>21), providing a clear perspective on the impact of mental conditions on patients’ lives [19].

Use of These Tools in the Study

These tools were used to provide a comprehensive and multidimensional assessment of the cognitive and affective functions of each participant. The MMSE was used for the initial assessment of cognitive function, the MoCA for detecting subtle cognitive dysfunctions, and the HADS for evaluating affective components. These assessments provided valuable data for further analysis, contributing to the understanding of the interactions between mental health and respiratory symptoms. 

### 2.4. Statistical Analysis

In the statistical analysis conducted for this study, we approached the data in successive stages to ensure rigor and depth in the interpretations. Initially, we used the Shapiro–Wilk test to evaluate the distribution of the data, ensuring that subsequent statistical methods were appropriate for our dataset.

After verifying normality, we applied the chi-square test and the Kruskal–Wallis test to identify significant differences between groups. These tests are essential in evaluating our initial hypotheses within the framework of classical analysis, providing a solid foundation for statistical conclusions. The *p*-values obtained from these tests highlighted significant differences, with a *p*-value < 0.05 considered an indicator of statistical significance. At this stage, we used SPSS software version 26.0 IBM Corporation, (Armonk, NY, USA), which is a standard in data analysis for medical research due to its advanced capabilities and ease of use.

Mean values ± standard deviation were calculated for continuous variables, while proportions were used to describe categorical variables, thus providing a clear perspective on the data distribution within the studied population. This allowed for a deeper interpretation of the influence of various variables on the study outcomes.

Due to the unclear results observed in the initial stage of classical analysis, we explored advanced clustering techniques using Python version 3.9. By applying the K-means method, we identified and grouped patients with similar profiles, which allowed for a more detailed and visual examination of the data. This machine learning approach revealed complex patterns and interdependencies between symptoms, enhancing our understanding of their interaction dynamics. The formed clusters were clearly defined by means and standard deviations.

## 3. Results

### 3.1. Demographic and Clinical Characteristics

The final cohort included 100 patients, with ages ranging from 20 to 87 years and an average age of 61 ± 11.9 years. The gender breakdown was 58% male and 42% female. The majority of patients (64%, *n* = 64) resided in rural environments. Employment data indicated that 26% of participants were actively employed, 8% were unemployed, and 66% were retired. Educational levels, assessed using the International Standard Classification of Education (ISCED), showed that 3% of patients fell into category one, 47% into category two, another 47% into category three, and 3% into category six. Body Mass Index (BMI) classifications revealed that 41% of the participants were obese (BMI > 30), 22% were overweight, 36% had a normal weight, and one was underweight.

Smoking status was reported as 39 current smokers, 23 former smokers, and 38 who had never smoked. Alcohol use was categorized with 11 participants being chronic users, 68 occasional users, and 21 abstainers.

The clinical profile included 23 patients with asthma, of whom nine had controlled asthma and 14 had partially or uncontrolled asthma. The remaining 77 patients were diagnosed with chronic obstructive pulmonary disease (COPD), categorized as 39 with severe stage IV GOLD, 19 with moderate stage III GOLD, and 19 with mild to moderate stages II/I GOLD. Additionally, twelve patients were under psychiatric care, with seven receiving medication for both anxiety and depression, one on anti-anxiety medication alone, and four on antidepressants.

### 3.2. Results of the Normality Test for Evaluative Measurement Instruments

Upon analyzing the results of the normality tests for each measurement instrument, we observed significant deviations from a normal distribution, as evidenced by very low *p*-values. (Table 2) This suggests variability or asymmetry in the data distribution for each instrument, potentially impacting the interpretation and subsequent analysis of the data.

For the cognitive evaluation tools (MoCA and MMSE), the non-normal results may reflect a distribution of scores that are either non-uniform or exhibit anomalies. This could influence the interpretation of the patients’ cognitive levels and might necessitate the use of non-parametric statistical methods for accurate data analysis.

Regarding the tools assessing states of anxiety and depression (HADS-A and HADS-D), the non-normal results suggest a diversity of symptoms among patients, with potential asymmetries in the distribution of these symptoms. Therefore, interpreting the levels of anxiety and depression might require a careful consideration of the data distribution and the individual characteristics of the patients.

For the COPD assessment tool (CAT) and the measurement of respiratory function (FEV1), the non-normal results may indicate varying disease severities and degrees of respiratory function impairment among patients. This variation could affect the evaluation and management of the disease and may necessitate specific statistical approaches for data analysis.

Given the non-normal results of the normality tests for each measurement instrument, we opted to use the Kruskal–Wallis Test for data analysis. This non-parametric test is well-suited for comparing three or more groups when the data are not normally distributed. By employing the Kruskal–Wallis Test, we can assess significant differences between patient groups for each measurement instrument without the assumption of a normal data distribution. Choosing this method allows us to obtain valid and interpretable results despite the deviations from normality in our data.

### 3.3. Analysis of the Association between MRC Scores and Cognitive and Affective Evaluations of Patients

**Figure 2:**
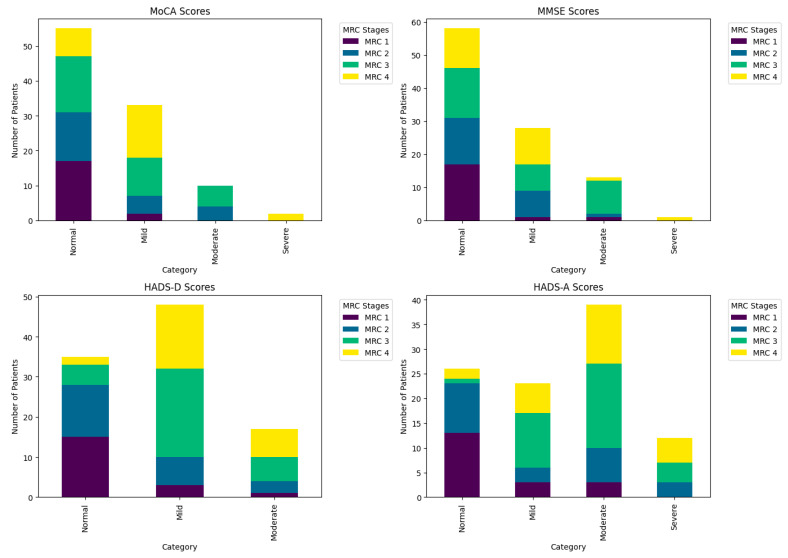
Cognitive and mental health scores grouped by MRC score.

The analysis of the MoCA scores reveals that 30.91% of patients with an MRC score of one (dyspnea on intense exertion) had normal MoCA scores, compared to only 14.55% of those with an MRC score of four (severe dyspnea at rest). (Figure 2) This indicates an increased prevalence of cognitive impairment as the severity of dyspnea intensifies (chi-square = 30.090, *p* < 0.001), suggesting a negative correlation between cognitive function and the severity of respiratory symptoms (Table 3).

The MMSE evaluations similarly reflect a pattern where 29.31% of patients with an MRC score of one had normal evaluations, decreasing to 20.69% for those with an MRC score of four, demonstrating a significant level of association (chi-square = 25.299, *p* = 0.003).

The analysis of the HADS-D (Hamilton Depression Scale) shows a clear link between the degree of dyspnea and depressive symptoms. Only 5.71% of those with severe dyspnea (an MRC score of four) were classified as normal, compared to 42.86% with an MRC score of one (chi-square = 35.322, *p* < 0.001), indicating that the severity of dyspnea is associated with more pronounced depressive symptoms. Similarly, HADS-A (Hamilton Anxiety Scale) scores reveal a lower prevalence of anxiety among patients with less severe dyspnea (50.00% normal at an MRC score of one), decreasing to 7.69% for those with severe dyspnea (an MRC score of four), with a statistically significant association (chi-square = 37.165, *p* < 0.001).

These results highlight a clear correlation between the severity of respiratory symptoms and the deterioration of mental state, both cognitive and affective.

### 3.4. Analysis of the Association between FEV1 Scores and Cognitive and Affective Evaluations of Patients

**Figure 3:**
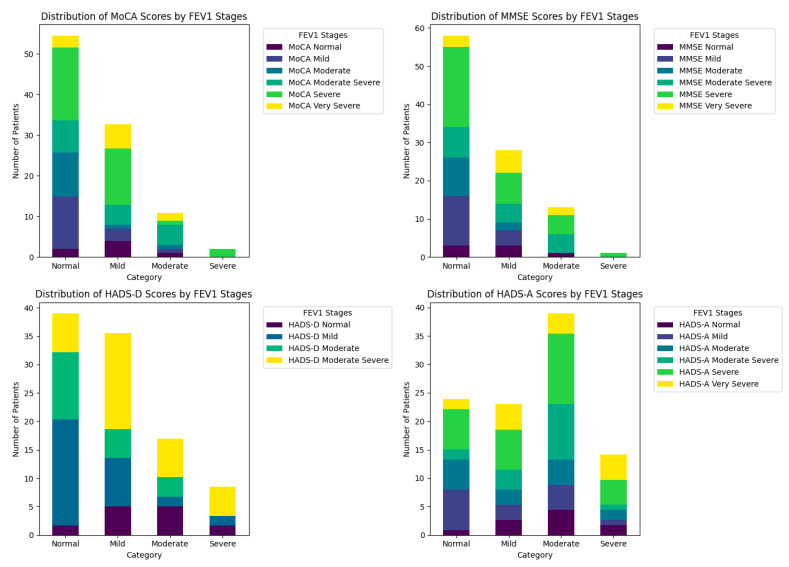
Cognitive and mental health scores grouped by FEV1 score.

The analysis of FEV1 scores, which assess the severity of airway obstruction, and performance on cognitive and affective tests shows varied distribution patterns (Figure 3).

The MoCA analysis reveals a varied distribution of scores across FEV1 severity grades, with 32.73% of patients with very severe FEV1 exhibiting normal MoCA scores, suggesting that cognitive impairment is not directly proportional to the severity of respiratory obstruction (chi-square = 27.652, *p* = 0.024). This indicates that additional factors may influence cognitive resilience in the face of respiratory challenges (Table 4).

MMSE results show a similar pattern, with the highest proportion of normal scores (36.21%) in the severe FEV1 category, and the lowest percentage of normal scores (5.17%) at the ends of the FEV1 severity spectrum, normal and very severe (chi-square = 18.244, *p* = 0.25). These results suggest a complexity in how cognitive function is affected by respiratory disease progression.

The distribution of HADS-D scores shows a correlation between the level of depression and FEV1 severity, where patients with moderate to very severe FEV1 exhibit higher percentages of scores indicative of depression. This may reflect the psychological impact of a more serious respiratory status (chi-square = 17.561, *p* = 0.063).

On the HADS-A scale, a significant association between anxiety and FEV1 is observed, with 30.77% of patients in the normal and very severe categories having normal scores, indicating a variable distribution of anxiety depending on FEV1 severity (chi-square = 28.734, *p* = 0.017). This could suggest that anxiety is influenced by the perception of health status and the uncertainty related to prognosis.

### 3.5. Analysis of the Association between CAT Scores and Cognitive and Affective Evaluations of Patients

In examining the relationship between scores on the COPD Assessment Test (CAT) and performance on cognitive and affective tests, a variety of distribution patterns emerged. The analysis of MoCA scores across CAT categories indicates that the highest proportion of patients with normal MoCA scores (38.18%) is observed in the high CAT category, followed by the very high CAT category (27.27%). This suggests a tendency for the preservation of a normal cognitive state even among patients experiencing more severe COPD symptoms (chi-square = 17.958, *p* = 0.036).

For the MMSE test, the distribution is relatively balanced across different CAT levels, with the most normal scores (36.21%) appearing in the high CAT category. This suggests that cognitive function may remain stable up to moderate levels of COPD severity, with a slight increase observed in the very high CAT category (31.03%), potentially indicating cognitive adaptation in the face of advancing COPD symptoms (chi-square = 10.578, *p* = 0.306).

The distribution of HADS-D scores shows a strong correlation between the degree of COPD severity and depressive manifestations. A significant proportion of patients with scores indicative of depression is observed in the very high CAT category (43.75%), indicating an increase in the incidence of depression as COPD severity progresses (chi-square = 31.990, *p* < 0.001).

On the HADS-A scale, patients in the medium CAT category have the highest proportion of normal scores (46.15%), but anxiety remains a significant concern across all categories, with an increasing trend observed in higher CAT categories, peaking at 41.03% in the very high category. This underscores an association between COPD severity and anxiety levels, indicating an increase in anxiety among patients with more severe forms of the disease (chi-square = 20.427, *p* = 0.015).

### 3.6. Exploration of the Interaction between MRC Scores and Numeric Evaluations of Cognitive and Affective Performance of Patients

In analyzing data according to MRC groups for cognitive and affective tests, a complex relationship between the severity of respiratory symptoms and the cognitive and affective performance of patients is observed (Table 5).

For the MoCA test, a progressive decline in cognitive performance is noted as dyspnea severity increases. The MRC 1 group shows relatively high cognitive stability (mean 26.16, SD ± 2.14, IQR of 1.0), while the MRC 4 group displays lower average scores (mean 22.68, SD ± 4.98, IQR of 5.0), indicating greater variability in cognitive responses among this group (*p* = 0.040).

MMSE results reflect a similar pattern, with higher average scores and smaller variation in the MRC 1 group compared to the MRC 4 group, which shows a lower average (mean 23.44, SD ± 4.07) and wider range of dispersion. Although variations between groups are not statistically significant (*p* = 0.141), the overall trend suggests deterioration in cognitive function as dyspnea severity increases.

HADS-D scores indicate an increase in depressive symptoms with worsening dyspnea. The MRC 1 group exhibits lower average depressive symptoms (mean 5.68, SD ± 2.65) compared to the MRC 4 group, which shows higher average symptoms (mean 10.24, SD ± 3.79). The differences between groups are statistically significant (*p* < 0.001), highlighting a strong correlation between the increase in respiratory symptom severity and depressive symptoms.

Similarly, on the HADS-A scale, a progressive increase in anxiety levels as dyspnea severity advances is observed. The MRC 1 group has a relatively lower average anxiety score (mean 7.47, SD ± 4.49) compared to the MRC 4 group, which shows higher anxiety (mean 14.28, SD ± 6.05). The differences between groups are significant (*p* < 0.001), suggesting more pronounced anxiety in patients with more severe dyspnea.

### 3.7. Exploration of the Interaction between FEV1 Scores and Numeric Evaluations of Cognitive and Affective Performance of Patients

Analyzing data across FEV1 groups for cognitive and affective tests reveals variations in performance and affective state relative to the severity of respiratory obstruction as measured by FEV1. For the MoCA test, the average scores and their dispersion (IQR) vary significantly between groups. The group with mild FEV1 has relatively high average scores (mean 25.24, SD ± 3.68) and reduced dispersion (IQR of 1.0), indicating stability in cognitive performance at this level of obstruction. In contrast, the moderate-severe FEV1 group shows lower average scores (mean 22.39, SD ± 5.23) and a large IQR (9.5), reflecting greater variability in cognitive responses. This contrast suggests that an increased severity of pulmonary obstruction may negatively impact cognitive capacity (*p* = 0.034).

MMSE trends are similar, with groups having mild and moderate FEV1 maintaining higher average scores (mean 25.35, SD ± 2.47 and mean 25.75, SD ± 1.86, respectively), while groups with moderate-severe and very severe FEV1 exhibit declines in performance, with a wide dispersion of scores (*p* = 0.043), underscoring the impact of respiratory dysfunction severity on cognitive function.

HADS-D analysis shows that depressive symptoms increase with FEV1 severity. Groups with normal and moderate-severe FEV1 present higher average depressive symptoms (mean 9.71, SD ± 3.73 and mean 9.33, SD ± 3.80), linking advanced respiratory dysfunction with an increase in depressive symptoms, though these differences are not statistically significant (*p* = 0.096).

For HADS-A, variability in scores is evident across all groups. The group with normal FEV1 shows the highest levels of anxiety (median 16.0, IQR 6.0), illustrating that anxiety can be predominant even in the early stages of pulmonary obstruction. This trend is reinforced by significant results from the Kruskal–Wallis test (*p* = 0.033), confirming the link between anxiety and the severity of respiratory impairment (Table 6).

### 3.8. Cluster Analysis—Identifying Patient Profiles Based on Cognitive, Affective, and Respiratory Characteristics

To deepen our understanding of latent structures and complex relationships between the variables studied, we adopted cluster analysis as a complementary method to traditional statistical analyses. Prior chi-square tests indicated significant differences between groups for categorical variables, suggesting the presence of distinct patterns in the data that could be further elucidated through clustering. Additionally, the Kruskal–Wallis test illustrated significant variations in the distributions of continuous variables across different categories, providing a robust foundation for a detailed exploration of how these variables naturally group together.

By implementing cluster analysis, we aimed to identify homogeneous groups within the dataset, reflecting essential differences between patients based on a complex set of cognitive, affective, and respiratory characteristics. This methodological approach, part of unsupervised machine learning techniques, allows us to go beyond initial findings and provide a much more nuanced perspective on the interactions and manifestations of the variables studied in the examined population. Cluster analysis decodes the internal structure of the data, facilitating the discovery of new patterns and correlations that were not immediately evident through conventional statistical methods.

We used the k-means clustering method due to its simplicity and computational efficiency, which allows for partitioning the data into k clusters by minimizing the internal variance in each cluster. K-means clustering is flexible and robust, allowing the selection of the number of clusters based on exploratory analysis and is capable of handling noise and outliers present in medical datasets.

Our process included data preprocessing for normalization, using StandardScaler to ensure that all variables contribute equally to the distances calculated between data points. We selected the optimal number of clusters using the “elbow” method, which analyzes the internal variance of the clusters as a function of the number of clusters. After this, we applied the k-means algorithm to partition the data and validated the resulting clusters using internal validation indices, such as the silhouette index, to assess cluster cohesion and separation.

**Figure 4:**
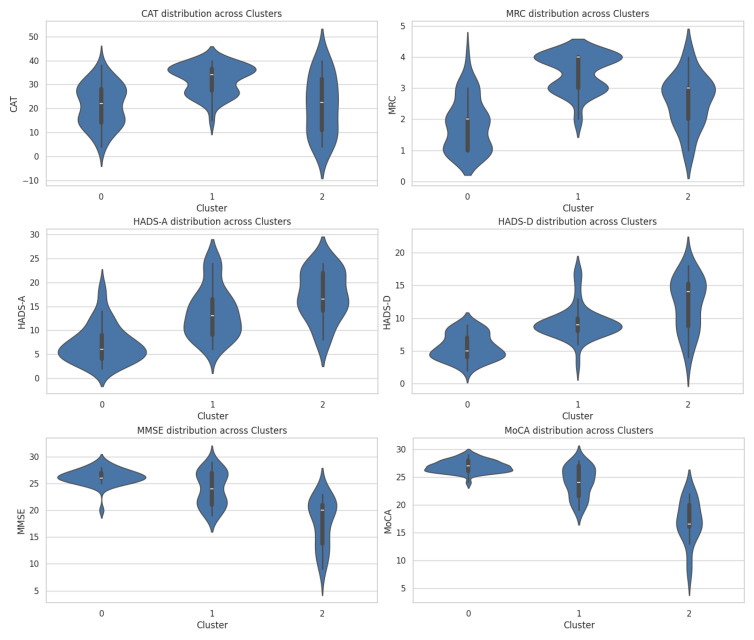
Comparison of cluster distributions using violin plots.

This machine learning technique enabled the discovery of new patterns and correlations that were not evident through traditional statistical methods, providing essential insights for personalizing the treatment and clinical management of patients. Following a rigorous implementation of cluster analysis on our dataset, we identified three main clusters, each characterized by specific attributes that outline distinct patient profiles (Figure 4).

These clusters suggest significant variations in how cognitive, affective, and respiratory characteristics associate and influence the overall state and management of disease among patients with chronic respiratory disorders (Table 7).

### 3.9. Characteristics of Cluster 0

Cluster 0 includes 50 patients and is characterized by profiles with moderate cognitive performance and moderate to high respiratory symptoms, indicative of intermediate stages of COPD. This grouping provides insight into the interaction between cognitive function and the impact of chronic respiratory diseases.

The average MoCA scores are 24.56 (SD ± 2.80), placing most patients in a zone of moderate cognitive function. The range of MoCA scores, from 19 to 29, indicates that some patients maintain normal cognitive function, while others experience mild cognitive difficulties.

HADS-D and HADS-A scores are 8.96 (SD ± 2.82) and 13.08 (SD ± 5.48), respectively. These values reflect moderate levels of depressive symptoms and anxiety, highlighting the psychological impact of respiratory conditions on patients in this cluster.

MRC scores in this cluster show a distribution where 27 patients (54%) have an MRC score of three, and 23 patients (46%) have an MRC score of four. This suggests that the majority of patients in this group experience severe respiratory symptoms, with a minority having very severe symptoms, underscoring the need for intense and possibly continuously monitored medical interventions to manage their respiratory condition effectively.

The average FEV1 score of 50.86 (SD ± 13.98) reflects impaired lung capacity, correlating with the level of respiratory symptom severity observed through MRC and CAT scores.

Given the profile of moderate cognitive performance and moderate to high respiratory symptoms, indicating a complex interaction between cognitive function and respiratory status, we have decided to name this cluster “Moderate Cognitive Respiratory.”

### 3.10. Identification Methods for Moderate Cognitive Respiratory Cluster

Patients in this cluster are identified through MoCA scores ranging from 20 to 25 and MMSE scores from 21 to 24, reflecting moderate cognitive impairment. HADS-D scores between seven and eleven and HADS-A scores between nine and sixteen indicate moderate levels of depressive symptoms and anxiety. MRC scores of three or four suggest moderate to severe respiratory symptoms. CAT scores ranging from 25 to 35 and FEV1 scores between 40 and 60 complete the profile of this cluster, characterized by moderate cognitive and respiratory impairment, necessitating balanced medical interventions (Table 8).

### 3.11. Characteristics of Cluster 1

Cluster 1 comprises 12 patients and is marked by significantly impaired cognitive and affective levels compared to other studied groups. The profile of this cluster reflects a profound impact of chronic respiratory disease on cognitive function and emotional state, highlighting a close interconnection between mental and respiratory health.

The average MoCA score for this cluster is 14.58 (SD ± 3.02), placing most patients in the category of major cognitive difficulties. This is significantly lower compared to other groups and indicates severely impaired cognitive function.

The average MMSE score is 16.50 (SD ± 4.62), confirming the presence of moderate to severe cognitive dysfunctions. The acute decline in these scores underscores the challenges patients face in managing daily life.

The average HADS-D score is 12.33 (SD ± 4.52), indicating a high level of depression in this cluster. This underscores a significant comorbidity between cognitive difficulties and depressive symptoms.

The average HADS-A score of 18.17 (SD ± 4.30) reflects a high level of anxiety, the highest among all groups, suggesting a state of constant unrest and an increased need for emotional support.

Reviewing the distribution of MRC scores in Cluster 1, we see that six patients (50%) have an MRC score of three, and two patients (16.67%) have an MRC score of four. This indicates that most patients in this cluster experience moderate to severe respiratory symptoms. Although four patients (33%) have an MRC score of two, indicating moderate symptoms, the significant presence of MRC scores three and four underscores that respiratory issues can considerably impact these patients’ overall state, likely exacerbating their cognitive and emotional difficulties further.

The average CAT score is 24.25 (SD ± 13.02), reflecting the impact of respiratory symptoms on patients’ quality of life, albeit to a moderate extent.

Given the significantly impaired cognitive and affective levels that highlight a tight interdependence between mental and respiratory health, we have decided to name this cluster “Severe Cognitive Respiratory.”

Identification Methods for Severe Cognitive Respiratory Cluster

The identification of patients in this cluster is based on MoCA scores below 20 and MMSE scores below 21, indicating severe cognitive impairments. HADS-D scores starting from 12 and HADS-A scores from 17 highlight high levels of depression and anxiety. MRC scores of three or four, predominantly four, and CAT scores between 20 and 30, along with FEV1 scores between 50 and 65, underline the severity of respiratory symptoms and the need for intense and specialized therapeutic interventions (Table 7).

### 3.12. Characteristics of Cluster 2

Cluster 2 consists of 38 patients and is notable for good cognitive performance and relatively mild respiratory symptoms, distinguishing it from the other two groups examined.

The average MoCA score is 25.84 (SD ± 2.62), placing most patients in a range of good cognitive functioning. This indicates an ability to adapt and manage daily activities effectively without major cognitive impediments.

The average MMSE score of 25.42 (SD ± 2.58) also confirms stable cognitive performance, demonstrating a good preservation of mental functions despite the presence of a chronic respiratory condition.

The average HADS-D score is 6.39 (SD ± 3.19), suggesting low levels of depressive symptoms among this group. This reflects effective emotional management or a minimal impact of health status on affective state.

The average HADS-A score of 8.03 (SD ± 5.16) also indicates a relatively low prevalence of anxiety, demonstrating a good capacity to adapt to health conditions and associated stress.

MRC scores indicate a mild severity of respiratory symptoms, with 19 patients (50% of the total cluster) having an MRC score of one. This suggests that respiratory symptoms do not constitute a major impediment in the daily lives of these patients.

The average CAT score is 21.61 (SD ± 9.58), reflecting a minimal impact of respiratory disease on daily life. This value confirms that the overall health status allows patients to maintain an active lifestyle.

Given the profile of stable cognitive performance and relatively mild respiratory symptoms, indicating an effective capacity to adapt and manage respiratory disease, we have chosen to name this cluster “Stable Cognitive Respiratory”.

### 3.13. Identification Methods for Stable Cognitive Respiratory Cluster

This cluster is distinguished by MoCA scores of 26 or higher and MMSE scores of 25 or more, indicating stable and efficient cognitive performance. Patients in this cluster exhibit HADS-D scores below seven and HADS-A scores below nine, suggestive of minimal affective symptoms. The prevalence of MRC scores of one or two and CAT scores of 25 or less reflects mild respiratory symptoms, allowing the maintenance of an active and minimally interventional lifestyle, with FEV1 scores of 55 or more confirming good pulmonary capacity (Table 7).

## 4. Discussion

This research delves into the intricate associations between respiratory symptom severity and cognitive as well as affective impairments among chronic respiratory disease patients, utilizing classic statistical analyses alongside clustering techniques. Motivated by the imperative to deepen understanding of the interactions between cognitive and affective dimensions with clinical presentations of respiratory diseases—a subject critical yet underexplored in current literature—the study identifies significant interdependencies through chi-square and Kruskal–Wallis tests. These patterns suggest a pronounced impact of respiratory symptom severity on patients’ cognitive functions and affective states, further elaborated through cluster analysis that delineates distinct patient subgroups with varied clinical needs.

Our mixed-method approach corroborates the clinical significance of cognitive and affective assessments in treating respiratory pathologies, underscoring the potential for tailored intervention strategies. The ensuing discussion integrates statistical and clustering analysis results, offering actionable recommendations aimed at enhancing patient management and quality of life.

### 4.1. Specific Results of Classic Analysis: Preliminary Data Evaluation

Our study’s chi-square tests have highlighted a clear correlation between the severity of respiratory symptoms, measured by MRC scores, and cognitive impairments, assessed through MoCA and MMSE tests. This association illustrates the direct impact of respiratory problems on cognitive function. As patients experience higher levels of dyspnea, their cognitive capacity appears to deteriorate. These findings emphasize that managing respiratory symptoms should include assessments and interventions aimed at mental health.

In the literature, Siciliano et al. addressed the discrepancy between objective and subjective cognitive measurements, highlighting the importance of carefully assessing cognitive function in the clinical context to accurately identify and treat cognitive issues associated with respiratory symptoms in non-demented Parkinson’s disease patients [20].

Kim et al. compared the efficacy of different MoCA protocols in detecting mild cognitive impairments, suggesting that both the complete and abbreviated versions of this test can be particularly useful in assessing patients with CNS tumors, demonstrating MoCA’s relevance in the context of neurological conditions and their impact on cognitive function [21].

Crișan et al. examined the relationship between frontal hypoxemia and cognitive impairment in COPD, observing that patients with severe COPD exacerbations exhibited significantly lower MoCA scores. This suggests a clear link between oxygen deficit and the deterioration of cognitive functions such as attention and language abstraction [22].

John et al. analyzed the impact of airway obstruction severity on cognitive function in COPD patients, finding that patients with severe airway obstruction performed poorly on cognitive tests such as the MMSE, which underscores the need for cognitive function monitoring in the management of COPD [23].

Finally, Villeneuve et al. investigated the prevalence and detection of mild cognitive impairment (MCI) in COPD, finding that a substantial proportion of patients with moderate to severe COPD had MCI, with predominant attention and executive dysfunctions. They noted that MoCA is superior to MMSE in detecting MCI, highlighting the necessity for regular cognitive assessments in the management of COPD [24].

Our study data show a significant link between the severity of dyspnea and the prevalence of depressive and anxious symptoms in patients. As respiratory symptoms worsen, patients report increased levels of depression and anxiety, thus underlining the importance of a psychosocial dimension in the management of chronic respiratory diseases.

This association is also supported by literature. Di Marco et al. observed that symptoms of anxiety and depression are frequently encountered in COPD patients, including those with mild forms of the disease. Interestingly, women reported higher levels of anxiety and depression compared to men, which directly affects the quality of life related to symptomatology [25].

Lou et al., in a study conducted in China, found that anxiety and depression were correlated with a higher BODE index, which assesses dyspnea severity. These conditions are associated with poorer health outcomes, indicating the importance of addressing these symptoms in the management of COPD [26].

An et al. investigated the validity of the BODE index as a predictor for anxious and depressive symptoms, concluding that this index is an effective tool for assessing COPD severity and can be used in clinical assessments [27].

Sharma et al. examined the relationship between dyspnea and the psychological state of COPD patients, showing that a significant proportion of patients report symptoms of anxiety (54.5%) and depression (51.6%), closely correlated with the perceived level of dyspnea. Their study emphasizes the need for including psychological assessments and interventions in the regular management of COPD [28].

In a similar context, Rantala et al. analyzed the link between exertional dyspnea and the overall impact of symptoms in patients with chronic respiratory failure. Their findings indicate that patients with severe dyspnea report higher scores for anxiety and depression, highlighting the necessity for a comprehensive evaluation of symptoms and appropriate treatment that addresses the extended burden of symptoms exacerbated by increasing dyspnea severity [29].

Our analyses using the Kruskal–Wallis test have highlighted significant variations in the distribution of cognitive and affective performance scores based on the severity of respiratory obstruction measured by FEV1. These variations underline the complexity of interactions between respiratory function and mental health, indicating that cognitive dysfunctions and affective issues are not uniformly distributed as respiratory disease progresses. The results suggest that additional factors, such as lifestyle or medical interventions, may influence these relationships.

These findings are contextualized by studies in the literature. For example, Athayde et al. evaluated different domains of the SGRQ respiratory questionnaire based on COPD severity levels. Their results indicated that there is no significant correlation between FEV1 and SGRQ scores, suggesting that the degree of airway obstruction does not uniformly reflect the impact on health status or cognitive and affective performance [30].

In another study, Sachdev et al. explored the relationship between lung function (FEV1 and forced vital capacity, FVC) and parameters of brain anatomy and cognitive function. They discovered that FEV1 is significantly negatively correlated with general brain atrophy and the ventricle-to-brain ratio (VBR) in men. Moreover, lung function was significantly correlated with information processing speed and fine motor dexterity, thus highlighting the relationship between reduced lung function and cognitive deterioration [31].

### 4.2. Cluster Analysis Results and Identified Profiles

#### 4.2.1. Analysis of the Moderate Cognitive Respiratory Cluster

This cluster comprises 50 patients with a moderate to high severity of respiratory symptoms and moderate cognitive performance. This grouping underscores the need for specific medical attention for the effective management of respiratory symptoms and monitoring of cognitive function.

#### 4.2.2. Moderate Cognitive Function and Correlation with COPD and Asthma

Within the Moderate Cognitive Respiratory cluster, the analysis of the complex interaction between cognitive function and respiratory symptoms in the context of chronic respiratory diseases such as COPD and asthma provides a deep insight into the neurobiological and pathophysiological mechanisms influencing this dynamic. Research highlights the fundamental role of cerebral hypoxia, systemic inflammation, and oxidative stress in cognitive decline associated with these conditions.

The study by Daulatzai emphasizes the impact of cerebral hypoxia on glucose hypometabolism, a key component in neurodegenerative pathology, including Alzheimer’s disease [32]. Additionally, research by Wiegman et al. in 2015 illustrates how oxidative stress can impair mitochondrial function, thus contributing to the progression of Alzheimer’s disease, highlighting the importance of this factor in cognitive deterioration [33]. Furthermore, a study by Zhang and colleagues in 2018 identifies inflammation as important in modulating cognitive decline, associating chronic inflammation with various cerebral pathologies, including Alzheimer’s disease [34].

Besides these biological mechanisms, longitudinal and cross-sectional studies reveal the impact of exacerbations of respiratory symptoms on cognitive function. An example is the study by Gong, Shang, and Wu, which demonstrates significant correlations between cognitive difficulties and dysfunctions in daily activities during acute COPD exacerbations [35], as indicated by studies by Dodd et al., which link these episodes to a poorer prognosis and longer hospitalization durations [36,37].

These findings highlight the necessity of the careful management of COPD symptoms to improve or at least maintain the cognitive function of patients. Therapeutic approaches such as long-term oxygen therapy and the administration of bronchodilators could provide significant benefits. The study by Karamanlı et al. shows that long-term oxygen therapy can mitigate cognitive deficit in COPD, suggesting a neuroprotective effect [38]. Concurrently, two studies by Lavoie et al. emphasize improvements in cognitive function through the use of bronchodilators in combination with physical exercise and behavioral modifications, demonstrating the benefits of an integrated approach [39,40].

Thus, introducing and optimizing oxygen therapy and bronchodilators in therapeutic regimens for patients with COPD can play a crucial role in preventing cognitive decline and improving their quality of life, suggesting a vital need for ongoing research and innovative therapeutic approaches.

#### 4.2.3. Variability in Cognitive Function

The Moderate Cognitive Respiratory cluster illustrates the complexity of the interaction between cognitive function and respiratory symptoms in the context of chronic respiratory diseases such as COPD and asthma. This cluster highlights the variability in cognitive function affected by demographic factors, lifestyle, and comorbidities, underscoring the importance of managing these factors to optimize clinical outcomes and the quality of life of patients.

According to the study by Hirano et al., the sedentary lifestyle of patients with COPD is associated with high levels of the pro-apoptotic proteins GDF-15, indicating a link between physical inactivity and cognitive deterioration [41]. Pierobon et al. identified advanced age and the presence of comorbidities as significant factors influencing adherence to physical exercises, highlighting the need for the adequate management of comorbidities to maintain cognitive function [42]. Furthermore, research by Incalzi et al. showed that a low level of education and lack of social support are linked to an increased prevalence of cognitive deficits, emphasizing the essential role of educational interventions and community support [43].

These findings underline the necessity of an active lifestyle, effective management of comorbidities, and educational and social support in maintaining and improving cognitive function. It is recommended to adopt integrated approaches that include physical, medical, and social interventions to enhance cognitive resilience.

Genetics also plays a crucial role in cognitive function in patients with COPD and asthma, influencing cognitive resilience through genetic predispositions. Studies on genetic variants, such as those coding for interleukins (IL-4, IL-6, IL-8, and TNF-α) and those studied by Reséndiz-Hernández and Falfán-Valencia, demonstrate how genetic polymorphisms affect cognitive function through mechanisms of oxidative stress and mitochondrial dysfunction [44]. Genetic signatures associated with type 2 inflammation, presented in the study by Christenson et al., suggest genetic links between asthma and COPD, which may influence the management and treatment of cognitive function [45].

The impact of comorbidities, such as hypertension and diabetes, on cognitive function is significant, with studies like those conducted by Hassing et al. and Dağ et al. showing links between physical health status and cognitive outcomes [46,47]. The effective management of these comorbidities is crucial for preventing cognitive decline and improving the quality of life.

In conclusion, these studies illustrate the complexity of the relationship between comorbidities and cognitive function, highlighting the need for a comprehensive approach in managing patients with COPD to improve both respiratory and cognitive outcomes.

#### 4.2.4. Psychological Impact of Respiratory Conditions

The psychological impact of chronic respiratory conditions like COPD is profound and complex, significantly influencing the psychological state and quality of life of patients. Studies have clearly linked the severity of respiratory symptoms to manifestations of anxiety and depression, which fluctuate with changes in respiratory status over both short and long terms.

Research by Doyle et al. demonstrates that anxiety and depression are closely linked to higher levels of fatigue and respiratory difficulties, and that functional capacity plays a crucial role in moderating this relationship [48]. According to Balcells et al., anxiety and depression significantly correlate with a decreased quality of life, especially in advanced stages of COPD, when the presence of other comorbidities can worsen the patient’s condition [49].

A study by Parreira et al. reveals that anxiety levels are moderately associated with emotional function and self-mastery among COPD patients, according to the Chronic Respiratory Questionnaire (CRQ) [50]. This underscores the importance of psychological assessment and intervention in managing COPD, suggesting that an integrated approach that includes both respiratory symptom management and psychological support is essential.

The predominant symptoms of COPD, such as dyspnea and reduced exercise capacity, significantly contribute to the development of anxiety and depression symptoms. Salerno and Carone have highlighted the impact of increased fatigue and decreased sleep quality, which are direct results of severe dyspnea and limitations in daily activities [51]. Additionally, Hill et al. have identified nicotine dependence as a factor that may exacerbate anxiety and depression among smokers with COPD [52].

Behavioral interventions and psychological support are vital in managing COPD. A study by Coventry et al. indicates the benefits of cognitive–behavioral therapy (CBT) in reducing symptoms of anxiety and depression [53]. Pulmonary rehabilitation programs, which combine physical exercises with health education, have proven effective in alleviating these symptoms.

Kew et al. emphasize that while non-pharmacological interventions are preferred, pharmacological treatments, such as long-acting inhalers, are essential for managing severe symptoms [54]. Sohanpal et al. have shown that personalized psychological support, including tailored cognitive–behavioral interventions, can significantly improve the management of anxiety and depression [55].

These findings highlight the need for an integrated approach in the treatment of COPD, combining psychological therapies with physical symptom management and lifestyle adaptations. Such an integrated approach can result in significant improvements in both the physical and mental well-being of patients, contributing to an enhanced quality of life and more effective management of chronic conditions.

#### 4.2.5. Severity of Respiratory Symptoms and the Need for Interventions

The effective management of severe respiratory symptoms in the context of COPD is crucial for improving the health outcomes of patients. Implementing evidence-based clinical guidelines and adapting to new assessment protocols are essential in this regard. A study by Jans et al. demonstrates that a comprehensive program adhering to guidelines improved pulmonary function and symptoms compared to usual care, underscoring the benefits of adopting clinical guidelines in medical practice [56].

A study by Witte et al. highlighted the advantages of a diagnostic care protocol that provides a holistic characterization of patients with COPD, improving the management of various aspects of patients’ lives, including quality of life and functional limitations [57]. Terasaki et al. illustrated how the use of electronic medical records (EMR) improved adherence to clinical guidelines for the assessment and management of stable COPD, demonstrating the effectiveness of such technological tools in enhancing the quality of care [58].

These studies illustrate the importance of adopting and adapting evidence-based clinical guidelines in medical practice to improve the management of severe respiratory symptoms. They also underline the role of technology and continuous education in enhancing adherence to clinical guidelines and optimizing health outcomes for patients.

Exploring innovative intervention techniques for COPD shows the potential of combined therapies and new approaches in improving the quality of life for patients. A study by Cui, Liu, and Sun highlighted the positive effects of comprehensive respiratory rehabilitation in combination with non-invasive positive pressure ventilation (NIPPV) on physical performance and quality of life in elderly patients with severe COPD [59]. Wedzicha et al. tested the effects of exercise training based on the severity of respiratory disability, noting improvements in health status and walking distance [60].

Daynes et al. investigated the use of a high-frequency oscillating device to improve dyspnea, with positive results in terms of maximum respiratory pressures and symptom reduction [61]. These findings highlight the potential of innovative and combined approaches in the treatment of COPD and asthma, with a direct impact on patients’ quality of life and respiratory function.

Integrated disease management programs, such as those studied by Kruis et al., which involve multiple treatment components administered by multidisciplinary teams, have shown significant improvements in health-related quality of life and exercise capacity, reducing the number of hospitalizations and hospital days [62]. These programs demonstrate the benefits of a long-term, integrated approach in managing COPD and asthma, underscoring the importance of multidisciplinary and continuous treatment.

Longitudinal studies emphasize the significant impact of interventions over time in managing COPD and reducing exacerbations, hospitalizations, and mortality, and demonstrate the necessity for ongoing monitoring and early interventions to prevent the deterioration of brain function and the acceleration of cognitive decline associated with cerebral hypoxia. These findings provide valuable insight into the interaction between pulmonary capacity and cognitive health, essential for effective and personalized treatment strategies in COPD.

#### 4.2.6. Impaired Pulmonary Capacity and Its Relationship with Cognitive Function and Emotional State

Research on the relationship between pulmonary capacity and emotional state highlights significant correlations between the severity of lung impairment and the prevalence of anxiety and depression symptoms in cases of chronic obstructive pulmonary disease (COPD), even at mild stages of impairment. Di Marco et al. observed that patients with COPD, regardless of the severity level, exhibit higher rates of anxiety and depression compared to the control group, with higher levels noted among women, independent of the degree of lung impairment measured by FEV1 [25].

Salerno and Carone point out that anxiety and depression are not necessarily directly related to the degree of pulmonary function impairment but rather to the presence of respiratory symptoms, where respiratory discomfort and reduced exercise capacity are the main contributors to anxiety and depression [51]. Krommydas et al. identified a significant correlation between decreased FEV1 and symptoms of depression among asthma patients, suggesting a biological link between depression and pulmonary function impairment [63].

It is essential to assess and manage mental health adequately among patients with COPD and asthma. The monitoring and appropriate treatment of anxiety and depression symptoms should be an integral part of managing these conditions to improve patients’ quality of life.

The impact of psychosocial and respiratory rehabilitation interventions on improving the emotional state of patients with reduced pulmonary capacity is significant. Güell et al. demonstrated that pulmonary rehabilitation could have positive effects on psychosocial morbidity and exercise capacity in patients with severe COPD, showing significant improvements in depression, hostility, chronic tension, and personality styles [64].

The study conducted by Pumar et al. evaluated the addition of cognitive–behavioral therapy (CBT) to a pulmonary rehabilitation program, without finding significant improvements in anxiety or depression symptoms, suggesting that the benefits of CBT might be limited by organizational factors such as slow recruitment and high dropout rates [65]. On the other hand, Basara et al. observed a significant decrease in levels of depression, anxiety, and stress, and a significant increase in the quality of life at the end of the intervention period in a pulmonary rehabilitation program [66].

These findings underscore the benefits of psychosocial and respiratory rehabilitation interventions and highlight the necessity of integrating these approaches into the comprehensive treatment of COPD and other chronic pulmonary diseases. Multidisciplinary approaches that combine pharmacological therapies with cognitive or emotional interventions can bring significant benefits. Studies that combine pulmonology with neuropsychology offer a valuable perspective on the complex relationship between pulmonary capacity and mental and cognitive health, highlighting the importance of an interdisciplinary and integrated approach in the care of patients with chronic respiratory conditions.

#### 4.2.7. Conclusions and Recommendations for the Moderate Cognitive Respiratory Cluster

The analysis of the Moderate Cognitive Respiratory cluster reveals that the interaction between moderate cognitive dysfunctions and the severity of respiratory symptoms, characteristic in the case of COPD and asthma, requires a careful approach. Optimizing pharmacological treatments, including oxygen therapy and bronchodilators, along with psychosocial support, is essential for preventing cognitive function decline and improving quality of life. Respiratory rehabilitation and psychological interventions help improve exercise capacity and maintain an active lifestyle, directly benefiting cognitive function. The careful monitoring of comorbidities, including cardiac conditions, plays a significant role in reducing exacerbations and effectively managing chronic conditions.

#### 4.2.8. General Characteristics of the Severe Cognitive Respiratory Cluster

The cluster includes 12 patients and is characterized by major cognitive difficulties and pronounced affective symptoms. Patients in this cluster exhibit low scores in cognitive tests and high scores for depression and anxiety, underscoring a tight interdependence between mental and respiratory states, necessitating complex and intensive therapeutic approaches.

#### 4.2.9. Severely Impaired Cognitive Function

Within the Severe Cognitive Respiratory cluster, studies emphasize a profound impact of chronic respiratory diseases on cognitive function and emotional state, illustrating a tight interconnection between mental and respiratory health. Clinical observations and research indicate that patients with COPD and asthma can experience a marked cognitive decline, associated with chronic hypoxia, systemic inflammation, and increased respiratory effort.

The study by Crișan et al. revealed that patients with COPD show significantly reduced MoCA scores, inversely correlated with inflammation markers such as C-reactive protein (CRP), fibrinogen, and erythrocyte sedimentation rate (ESR). These findings suggest that systemic inflammation and hypoxia contribute significantly to the cognitive impairment observed in these cases [22].

Incalzi et al. demonstrated that cognitive decline is accelerated in cases of severe bronchial obstruction, associated with worsening affective states. This indicates that patients with reduced pulmonary functions experience a more pronounced decline in cognitive function, which is linked to the severity of hypoxemia [43].

Studies by Wen et al. and Guo et al. highlight the negative impact of hypoxemia on cognitive function, even in cases of chronic asthma in children, affecting learning and memory capacities [67,68]. This underscores the need for integrated management that addresses both pulmonary and neuropsychological components in treating these conditions, to prevent or mitigate cognitive decline.

Addressing the complexity of cognitive deterioration associated with chronic respiratory diseases often involves a combination of pharmacological treatments, behavioral interventions, and technological solutions. Wang et al. explored the effectiveness of interventions based on physical and cognitive exercises, showing significant improvements in overall cognitive function, executive function, and delayed memory, suggesting that physical exercise can provide remarkable benefits [69].

Moreover, Ströhle et al. evaluated the effectiveness of pharmacological therapies versus physical interventions for improving cognition in individuals with Alzheimer’s and mild cognitive impairment, discovering that physical interventions can have a moderate to strong effect on cognitive function in Alzheimer’s cases and a lesser effect in mild cognitive impairment [64,70].

These findings highlight the necessity for a holistic and integrated treatment that combines pharmacological therapies with behavioral and physical interventions to maximize outcomes in improving cognitive function in patients with cognitive impairments or dementia. Integrated approaches, combining physical exercise with cognitive therapy, are essential for the effective management of chronic conditions, emphasizing the importance of a comprehensive strategy in the care of patients with COPD and other chronic pulmonary conditions.

#### 4.2.10. Impact of Cognitive Dysfunctions on Daily Life

Cognitive dysfunctions associated with chronic respiratory conditions such as COPD and asthma represent a significant barrier in maintaining an independent and active life for patients. Studies by Yazar et al. and Antonelli-Incalzi et al. demonstrate how cognitive deficits negatively impact patients’ ability to engage in daily activities, highlighting a tight correlation between low levels of blood oxygenation and cognitive decline [71,72]. Assessments used tools such as the Mini-Mental State Examination (MMSE) and the Clock Drawing Test, showing that patients with low scores on these tests also had limited autonomy.

These findings underscore the urgent importance of an integrated approach in diagnosis and treatment, which should include not only the management of physical symptoms but also the evaluation and support of cognitive functions. Such an approach could significantly improve the quality of life for patients by supporting their independence and capacity to participate in social life.

A crucial aspect in managing this interdependence is the implementation of adaptive support strategies. According to the study by Garbisson et al., online cognitive adaptation programs for caregivers can facilitate the selection of specific cognitive-adaptive strategies, which help improve daily functioning and reduce symptoms [67]. These programs may include adaptations of the home environment, support for family and caregivers, as well as integrating patients into community activities, all contributing to maintaining an adequate level of independence [73].

In addition to adaptive support, cognitive training, evaluated through the systematic review conducted by Bahar-Fuchs et al., has demonstrated positive effects on global cognition and verbal semantic fluency, offering medium-term benefits [74]. These non-pharmacological interventions can be extremely valuable in improving cognitive functions and supporting patients to maintain as normal a quality of life as possible.

Therefore, it is vital to place greater emphasis on personalized and comprehensive approaches in the treatment of patients with severe cognitive dysfunctions in the context of chronic respiratory diseases. Integrated interventions, combining pharmacological treatments with behavioral and support strategies, are essential to support independence and quality of life for patients, helping them to remain as active and integrated in their community as possible.

#### 4.2.11. Anxiety and Its Impact on Disease Management

In the Severe Cognitive Respiratory cluster, anxiety plays a significant role in how patients with COPD and asthma manage their treatments, significantly influencing adherence to prescribed medical regimens and overall health-related behaviors.

A meta-analytic study led by DiMatteo extensively explored the impact of anxiety and depression on patients’ noncompliance with medical treatment [75]. The findings revealed that depression is a significant risk factor for nonadherence, with depressed patients showing a threefold higher rate of nonadherence compared to non-depressed individuals. Anxiety, while having more variable effects, also affects treatment adherence, underscoring the need for specialized therapeutic interventions that address these mental health issues.

Furthermore, a study by Yorke et al. assessed the effectiveness of non-pharmacological interventions in adults with asthma [76]. This study included relaxation therapies, mindfulness, biofeedback, and cognitive–behavioral therapies (CBTs). The results indicated a consistently positive impact on asthma-related quality of life, psychological outcomes, and even pulmonary function in the case of relaxation therapy. These findings highlight the importance of psychological and stress management approaches in improving patient health.

Thus, these research findings underscore the critical need to evaluate and treat anxiety in managing COPD and asthma. Considering the profound impact of anxiety on health-related behavior and treatment adherence, it is essential to integrate medical interventions with psychological ones to provide significant improvements in managing these complex conditions. This involves not only managing respiratory symptoms but also actively supporting patients psychologically, ensuring a comprehensive approach that contributes to the overall improvement of their quality of life.

#### 4.2.12. Quality of Life and Respiratory Symptoms—Interaction with Cognitive and Emotional State

In the Severe Cognitive Respiratory cluster, studies emphasize that the severity of respiratory symptoms in patients with COPD and asthma profoundly affects not only their respiratory function but also their cognitive and emotional states. This demonstrates the need for an integrated approach in treatment, which should include not just the management of physical symptoms but also evaluation and interventions for mental health.

A study by Ţîrcă et al. explored the impact of anxiety disorders on asthma management, finding that anxiety affects symptom perception and treatment adherence [77]. This leads to noncompliance, compromising treatment success and patients’ quality of life, which underscores the importance of integrating mental health treatments into care plans.

Additionally, research conducted by Dodd et al. evaluated the utility of the COPD Assessment Test (CAT) in measuring the impact of symptoms during exacerbations [78]. The results indicate that elevated CAT scores reflect the severity of exacerbations, determined by pulmonary function and their duration. This highlights the need for a holistic approach in treatment, including regular assessments and mental health interventions.

These findings emphasize the need for implementing integrated and personalized care, which should involve collaborations between pulmonologists, psychiatrists, and psychologists to ensure the effective and comprehensive management of COPD and asthma. This would improve both the physical and emotional states of patients.

Recent studies on the relationship between the severity of respiratory symptoms and quality of life use assessment tools such as the CAT score to provide a detailed perspective on the impact of patients’ health conditions. For example, a study by Herrero et al. explored the influence of the CAT score on clinical decisions, such as the decision to hospitalize patients with COPD exacerbations in emergency departments [79]. The results showed that high CAT scores are associated with an increased likelihood of hospitalization, reflecting the severity of respiratory symptoms and their impact on quality of life.

In another study, Ayora et al. analyzed the psychometric parameters of two quality of life questionnaires, SGRQ and CAT, in assessing patients hospitalized for COPD exacerbations [80]. The study revealed differences in the difficulty of completing and scoring the questionnaires and identified significant correlations between decreased health-related quality of life and symptoms such as dyspnea and dependence on others in daily activities.

These studies highlight the complexity of assessing the impact of respiratory symptoms on quality of life and the necessity of using detailed and tailored assessment tools. They also indicate the importance of integrated approaches in the treatment of COPD and asthma, which should include regular evaluations and interventions for mental health, to improve both the physical and emotional well-being of patients. These integrative approaches are essential for the effective management of these complex conditions.

#### 4.2.13. Strategies for Enhancing Quality of Life

Within the Severe Cognitive Respiratory cluster, the impact of chronic respiratory disease on cognitive function and emotional state highlights the importance of a systemic approach in treatment. It is vital to integrate not only the management of physical symptoms but also the necessary evaluations and interventions for mental health.

A key aspect in improving the quality of life for these patients is the personalized use of respiratory rehabilitation and assistive technologies. The study by Ji et al. illustrates the effectiveness of a tailored pulmonary rehabilitation program, which included interactive physical training and monitoring through mobile applications, achieving positive results in improving the distance covered in the six-minute walk test (6MWD) and the overall quality of life of patients [81]. This demonstrates the benefits of integrating mobile technology into rehabilitation programs for the efficient management of respiratory symptoms.

Moreover, the use of assistive technologies, such as mobile applications for health monitoring, has facilitated more effective self-management of symptoms and improved patient independence. These technologies allow for real-time monitoring and reporting of health data, thus contributing to the personalization and continuous adaptation of treatment.

To meet the complex needs of these patients, the adoption of integrated and personalized approaches is essential. Interdisciplinary collaboration in the care of patients with COPD underscores the need for close cooperation among pulmonologists, psychiatrists, psychologists, and physiotherapists to provide comprehensive and tailored care.

The study conducted by Rivas et al. within the NHS in the UK has highlighted that multidisciplinary peer reviews improve collaboration and reconcile different perspectives among specialties, which contributes to enhancing the quality of care provided to patients with COPD [82]. Additionally, research by Nurmatov et al. showed that holistic interventions that include direct patient feedback can lead to significant improvements in their quality of life, emphasizing the importance of involving patients in the care process [83].

Thus, implementing an integrated and interdisciplinary approach can lead to significant improvements in quality of life and the efficient management of COPD, demonstrating the effectiveness of collaboration among various medical specialties and the integration of patient feedback in the ongoing evaluation and improvement of care strategies. This approach not only improves the physical condition of patients but also their emotional state, contributing to a better quality of life.

#### 4.2.14. Conclusions and Recommendations for the Severe Cognitive Respiratory Cluster

Patients in the Severe Cognitive Respiratory cluster, characterized by major cognitive difficulties and pronounced affective symptoms, require integrated interventions that combine pharmacological therapies with behavioral and physical support. Approaches that combine physical exercises with cognitive therapy and psychological support are vital for the effective management of these conditions. These strategies improve not only the physical symptoms but also the social independence of patients, contributing to a better quality of life. Treating anxiety and depression is fundamental, given the significant impact of these conditions on health-related behavior and treatment adherence.

#### 4.2.15. General Characteristics of the Stable Cognitive Respiratory Cluster

The Stable Cognitive Respiratory cluster, composed of 38 patients, exemplifies the crucial role of cognitive performance in the effective management of chronic diseases. This cluster is distinguished by good cognitive scores, such as an average MoCA score of 25.84 and an average MMSE score of 25.42, indicating stable cognitive ability and effective adaptation to health conditions. This capacity to manage daily activities despite chronic respiratory conditions is essential for maintaining a high quality of life.

The study by Tiffin-Richards et al. emphasizes the importance of cognitive assessment in the care of patients with complex chronic conditions, demonstrating that higher cognitive scores are associated with better adherence to treatment [84]. This association is vital for optimizing the treatment and support provided to patients with chronic renal failure, suggesting similar benefits for other chronic conditions such as COPD.

Although the study by Dulohery et al. did not observe a direct correlation between cognitive function and self-management or quality of life in COPD patients, its results underscore the need to continue exploring the links between cognitive function and disease management [85]. Integrating cognitive assessment into care protocols is essential for addressing the complexity of managing chronic diseases, not only through medication but also by supporting cognitive function maintenance.

Health education and psychosocial support also play key roles in improving the management of chronic health conditions. The study by Mowbray et al. on supportive education programs for adults with psychiatric disabilities illustrates how these programs can enhance social integration and the achievement of personal and professional goals [86]. These programs are important for improving access to education and community integration for people with mental health conditions.

Additionally, research by Barlow and Ellard has highlighted the effectiveness of psychosocial interventions for children and adolescents with chronic illnesses [87]. Cognitive behavioral techniques used in these interventions improve self-efficacy, disease management, family functioning, and psychosocial well-being, underscoring the importance of integrating these interventions into the management of chronic diseases. These approaches contribute to improving the autonomy and independence of patients, facilitating adaptation to the restrictions imposed by chronic health conditions and enhancing overall quality of life.

These findings and educational and support approaches, integrated into the management of the Stable Cognitive Respiratory cluster, underline the importance of cognitive performance and effective adaptation in improving the quality of life of patients with chronic conditions, providing a robust framework for ongoing treatment and support.

#### 4.2.16. Preserving Cognitive Function in the Presence of Respiratory Conditions

Preserving cognitive function in the context of respiratory conditions is crucial, given the significant impact of these conditions on the quality of life of patients. Current studies, such as those conducted by Sarınç Ulaşlı et al., underscore the relationship between the severity of bronchial obstruction and the decrease in MMSE scores, illustrating how severe COPD can negatively affect cognitive function [88]. This highlights the importance of monitoring cognitive function and prompt interventions to counteract cognitive deterioration associated with frequent disease exacerbations.

At the same time, the study by Huang et al. explores the impact of malnutrition on cognitive function, showing a negative correlation between MMSE scores and nutritional risk [89]. This emphasizes the need for nutritional assessments and interventions as an integral part of managing chronic respiratory diseases, underscoring malnutrition as an independent risk factor for cognitive decline.

An integrated management that includes periodic assessments of cognitive function, as well as personalized interventions, becomes indispensable. Strategies that integrate nutritional risk management and promote a healthy lifestyle are fundamental for maintaining and enhancing cognitive resilience in patients with chronic respiratory conditions. The systemic approach in managing COPD and asthma strongly focuses on the interdependence between physical and cognitive health.

The study by Troosters et al. demonstrates that health education and psychosocial support can improve patients’ ability to manage COPD, by enhancing treatment adherence and reducing the frequency of exacerbations [90]. Educational programs that include stress management and self-care skills development have a significant positive impact on the quality of life of patients.

Furthermore, Emery et al. evaluated the effects of a physical rehabilitation program on the cognitive function and psychological state of patients with COPD [91]. The results indicate notable improvements in cognitive performance and a reduction in symptoms of anxiety and depression, highlighting the importance of physical exercises within a comprehensive management of COPD.

These studies confirm the necessity of an integrated approach that includes pharmacological treatments, health education, psychosocial support, and physical activities, contributing to improving both physical and cognitive health states. Implementing these components can facilitate a general enhancement of life quality for patients, thereby strengthening resilience and adaptive capacity in the face of chronic respiratory conditions.

#### 4.2.17. Reduced Impact of Depressive Symptoms

The reduced impact of depressive symptoms in the Stable Cognitive Respiratory cluster is a central aspect that underscores the benefits of the effective management of chronic respiratory conditions such as COPD and asthma in the context of mental health. Current studies highlight the role of lifestyle factors and social support in reducing depressive symptoms. For instance, the research by Xu et al. explored how sleep quality, sleep duration, regular physical activity, sun exposure, and daily breakfast consumption can influence a decrease in depression scores among students, identifying these healthy lifestyle elements as effective protective factors against depression [92].

Moreover, the study by Mata et al. examined the effect of acute physical exercises on individuals who have recovered from depression and found that physical activity mitigates the negative effects of repeated sadness induction [93]. Participants who engaged in acute physical activities reported a smaller increase in negative affect compared to those who did not exercise, suggesting that physical exercise can serve as a protective shield against the recurrence of depressive symptoms, particularly in situations of repeated emotional stress.

These findings emphasize the importance of early interventions, including social support and physical exercises, as essential ways to maintain cognitive and emotional health for individuals with chronic conditions. Implementing preventive and proactive strategies can play a crucial role in reducing the risk and severity of depression in these populations. Studies in the field of psychosocial and emotional interventions, such as those conducted by Thompson et al. and Kuyken et al., have demonstrated the effectiveness of mindfulness-based cognitive therapy (MBCT) in the emotional management of patients with chronic conditions [94,95]. The UPLIFT project, for example, showed that MBCT intervention, administered via web or phone, significantly reduces the incidence of major depressive episodes in people with epilepsy, simultaneously improving knowledge, skills, and satisfaction with life.

Compared to active control conditions for relapse prevention in patients with recurrent depression, MBCT has proven to be as effective as maintenance medication in reducing the risk of relapse. This underscores the utility of MBCT as a potent non-pharmacological intervention and its relevance in integrating psychosocial support into medical care plans for patients with COPD and asthma, offering effective strategies for improving emotional management and reducing depressive symptoms.

Thus, these pieces of evidence suggest that an integrated approach that encompasses psychosocial support, healthy lifestyle strategies, and psychotherapeutic interventions can significantly contribute to improving the quality of life and stabilizing cognitive function and emotional state among patients in the Stable Cognitive Respiratory cluster.

#### 4.2.18. Anxiety and Adaptation to Illness

Within the Stable Cognitive Respiratory cluster, studies on the impact of anxiety on the management of chronic diseases such as COPD underline that low levels of anxiety can facilitate better adaptation to illness conditions. Dowson et al. used the Hospital Anxiety and Depression Scale (HADS) to assess anxiety and depression among COPD patients during recovery, observing that lower scores on the HADS anxiety subscales are associated with better adaptation to treatment and an improvement in health status throughout rehabilitation [96]. This highlights the crucial role of anxiety management in improving health outcomes.

Preventive interventions, such as cognitive–behavioral therapy, have also been evaluated by Puhan et al. to prevent severe depressive symptoms among patients with chronic conditions [97]. The study results indicate that early and proactive interventions can significantly reduce symptoms of anxiety and depression, contributing to better adaptation to illness and improving the quality of life.

These findings underscore the importance of managing anxiety in enhancing patients’ adaptation to chronic illness conditions and in facilitating better adherence to treatment and symptom management, suggesting that effective anxiety control can have a significant impact on overall health management. Psychosocial support plays a crucial role in reducing anxiety and improving the quality of life for patients with chronic conditions, such as cancer and hematological disorders. The study by Weis shows the benefits of support groups for cancer patients, offering not only information and emotional support but also stress management strategies and relaxation techniques [98]. These groups have proven to be beneficial not only to patients but also to their family members, alleviating disease-related stress.

Furthermore, research by Mackensen et al. highlights a discrepancy between the perceived needs for psychosocial support by health professionals and its utilization by patients with inherited bleeding disorders, underlining an underutilization of these services [99]. This indicates a heightened need for more accessible and well-informed psychosocial support programs.

Implementing well-structured psychosocial support programs can play a significant role in enhancing patients’ well-being and overall quality of life. Support groups, as demonstrated by the study by Weis, provide a valuable framework where participants benefit from emotional and social support, learning stress management strategies and problem-solving techniques [98]. This contributes to better adaptation to illness and reducing feelings of isolation, having a direct impact on the quality of life of cancer patients.

On the other hand, psychosocial interventions, such as those analyzed by Michalsen et al. in stress management for patients with heart diseases, demonstrate that mindfulness techniques and stress management not only improve quality of life but also positively influence the physical and psychological health of patients [100]. Stress reduction and enhanced emotion management are crucial for improving patients’ adaptation to illness.

Implementing such psychosocial support programs is crucial in the integrated management of chronic diseases, significantly contributing to enhancing quality of life and reducing anxiety, enabling patients to more effectively face daily health challenges.

#### 4.2.19. Mild Respiratory Symptoms and Their Impact on Daily Life

Within the Stable Cognitive Respiratory cluster, characterized by good cognitive performance and relatively mild respiratory symptoms, a direct correlation between the severity of respiratory symptoms and their impact on daily life is observed. The study conducted by Viramontes and O’Brien utilized the Short Form 36 (SF-36) and the Medical Research Council (MRC) classification to assess health-related quality of life among patients with chronic pulmonary diseases [101]. The results demonstrated that patients with milder symptoms can maintain a better level of physical activity and superior quality of life.

Furthermore, research by Mirdamadi et al. investigated the link between cardiopulmonary exercise tests and quality of life, using the St. George’s Respiratory Questionnaire (SGRQ) [102]. The study showed a correlation between the severity of respiratory dysfunction and quality of life, indicating that patients with milder respiratory symptoms experience better physical performance and an improved quality of life.

These findings underscore the importance of effective symptom management to allow patients with chronic diseases to actively participate in social life and maintain their performance at work, thus contributing to the overall improvement of quality of life. Early interventions, such as physical exercise programs and respiratory management therapies, play a crucial role in maintaining a low severity of symptoms and preventing health deterioration in patients with respiratory conditions. The study by Mendes et al. examined the effects of aerobic training on eosinophil inflammation and exhaled nitric oxide levels (FeNO) in patients with moderate or severe persistent asthma [103]. The results indicated significant reductions in sputum eosinophil counts and FeNO levels, suggesting improvements in asthma control and a reduction in exacerbations. This demonstrates that aerobic training can serve as an effective adjunct therapy in asthma management.

Additionally, a meta-analysis conducted by Lu et al. evaluated the impact of home-based breathing exercises on lung function, respiratory muscle strength, exercise capacity, dyspnea, and quality of life in patients with COPD [104]. The results from the included studies showed significant improvements in all of these areas, confirming the importance of breathing exercises as part of home-based pulmonary rehabilitation programs.

These findings highlight the efficacy of early and tailored interventions in maintaining health and quality of life for patients with mild respiratory symptoms, as well as in preventing the worsening of symptoms. Thus, patients in the Stable Cognitive Respiratory cluster benefit from effective adaptation to health conditions, with the opportunity to maintain an active and satisfying lifestyle.

#### 4.2.20. Reduced Impact of Respiratory Symptoms on Quality of Life

In the Stable Cognitive Respiratory cluster, characterized by good cognitive performance and mild respiratory symptoms, studies demonstrate that effective symptom management can have a significant positive impact on quality of life. The study by Dodd et al. used the COPD Assessment Test (CAT) to measure the impact of symptoms in pulmonary rehabilitation programs, finding improvements in CAT scores post-rehabilitation, which indicates an improvement in general health perception and the ability to perform daily activities [78].

Conversely, the research by Herrero et al. highlighted that high CAT scores during COPD exacerbations are associated with hospitalization decisions, emphasizing the importance of continuous monitoring and proactive management to prevent severe exacerbations that can negatively affect quality of life [79]. This underscores the necessity of maintaining symptoms at a low level to avoid complications that can impact patients’ independence and well-being.

The early implementation of appropriate interventions is essential for the effective management of chronic respiratory diseases. Sansores et al. demonstrated that universal COPD screening among smokers could be more effective than symptom-based strategies, showing the benefits of early detection and interventions to prevent disease progression [105].

Additionally, a meta-analysis by Cannon et al. evaluated the impact of self-management interventions on patients with COPD. The results showed significant improvements across all domains of the St. George’s Respiratory Questionnaire (SGRQ) and the six-minute walk test, with health education identified as the most effective component [106]. This underscores the important role of education in improving quality of life and physical capacity.

Preventive and early interventions, including universal screening and self-management strategies, are crucial for maintaining a minimal impact of respiratory symptoms and preventing the deterioration of health. To gain a comprehensive understanding of the dynamics of respiratory symptoms and their impact on quality of life, examining longitudinal and comparative research is essential. The multisite longitudinal study by Yorke et al. examined health-related quality of life (HRQoL) and symptomatology in patients with pulmonary hypertension, showing that higher levels of symptoms consistently negatively impact HRQoL [107].

These findings highlight the efficacy of longitudinal and comparative approaches in assessing the effects of early interventions and prevention strategies on the management of respiratory symptoms and improving quality of life, providing valuable insights for the effective management of chronic respiratory diseases. Implementing these approaches can facilitate more efficient symptom management and reduce the need for more intensive long-term treatments, contributing to the improvement of the overall health status and quality of life of patients in the Stable Cognitive Respiratory cluster.

### 4.3. Conclusions and Recommendations for the Stable Cognitive Respiratory Cluster

The Stable Cognitive Respiratory cluster illustrates the benefits of good cognitive performance and mild respiratory symptoms. Preventive and health maintenance interventions that enhance self-efficacy and disease management are fundamental for this group. Integrating cognitive–behavioral techniques into the treatment regimen helps improve autonomy and adaptation to health restrictions. Adequate pharmacological treatments, along with health education and psychosocial support, facilitate significant improvements in quality of life. Longitudinal studies evaluating the effects of these early and preventive interventions are crucial to confirm their long-term benefits in maintaining health and quality of life.

### 4.4. Future Research

#### 4.4.1. Discussion of Implications

The results of this study highlight the complex associations between the severity of respiratory symptoms and cognitive and affective impairments in patients with chronic respiratory diseases. Using cluster analysis, we identified distinct subgroups of patients, each with varying clinical needs. These findings suggest that a personalized treatment approach, considering both cognitive and affective aspects as well as the severity of respiratory symptoms, could significantly improve the quality of life for patients.

Cluster analysis enabled the identification of new patterns and correlations that were not observed through conventional statistical methods. Consequently, the results underscore the necessity of including cognitive and affective evaluations in the management of chronic respiratory diseases to develop more tailored intervention strategies that meet the individual needs of patients.

#### 4.4.2. Research Suggestions

For future research, we suggest conducting longitudinal studies to assess how the identified clusters change over time and how they influence disease progression and treatment response. Additionally, clinical trials should be conducted to test the effectiveness of personalized treatments based on these clusters. It would be beneficial to investigate the impact of personalized interventions on the cognitive function and affective state of patients within the context of managing respiratory symptoms.

#### 4.4.3. Practical Applications

Our findings can be implemented in practice by developing clinical guidelines that consider the different patient profiles identified. These guidelines could include specific recommendations for managing respiratory symptoms, monitoring cognitive function, and providing appropriate psychological interventions. For example, patients in clusters with moderate cognitive impairments and severe respiratory symptoms might benefit from combined therapies that include advanced respiratory treatments, cognitive therapies, and psychosocial support.

Implementing these findings could also involve using mobile technologies for the continuous monitoring of patients’ conditions and real-time treatment adjustments. Furthermore, respiratory rehabilitation programs could be personalized to include specific physical and cognitive exercises tailored to the needs of each patient subgroup.

## 5. Study Limitations

While the findings of our study are promising and represent a significant step forward in personalized treatment strategies for chronic respiratory conditions, it is important to acknowledge the limitations that might affect the broader application of our results. Data collection was confined to a single institution, the Leamna Pulmonology Hospital, which may limit the generalizability of our findings to other geographic or institutional contexts. Additionally, the demographic and clinical homogeneity of our study population could restrict the applicability of our results in different clinical or cultural environments.

Despite these limitations, the application of advanced machine learning techniques allowed us to overcome some of the typical constraints associated with small sample sizes. Machine learning’s ability to uncover complex patterns and subtle interactions within limited datasets significantly extends the insights that can be derived, compared to traditional statistical methods. The specialized literature supports the efficiency of machine learning even in scenarios with a small number of subjects, demonstrating its capability to operate effectively in ‘large p, small n’ conditions [108].

In summary, the methodology based on machine learning adds significant value, enabling a more detailed and personalized interpretation of data. This underscores the potential for future, more extensive and diverse studies to validate and expand the applicability of our findings. This section of our work exemplifies how data technology innovation can transform the management of complex health conditions, paving new pathways for improving patient care.

## 6. Conclusions

Our study employed advanced clustering techniques based on machine learning to investigate the interactions between the cognitive, affective, and respiratory profiles of patients with COPD and asthma. This innovative approach not only facilitated the identification of distinct clusters but also led to the development of a methodological framework that can be practically applied to tailor treatments based on the unique attributes of each identified cluster.

Patients within the Moderate Cognitive Respiratory cluster, who exhibit moderate cognitive dysfunctions and evident respiratory symptoms, could benefit from an integrated treatment regimen that includes oxygen therapy and bronchodilators, supplemented by psychosocial support and respiratory rehabilitation programs. Such interventions are anticipated to yield improvements in cognitive function and quality of life, as corroborated by similar studies in the literature.

Conversely, the Severe Cognitive Respiratory cluster, characterized by severe cognitive impairments and pronounced affective issues, necessitates a comprehensive therapeutic strategy that combines medications for respiratory symptoms with psychological and physical interventions. This integrated approach is aimed at enhancing daily functioning and overall patient well-being.

Meanwhile, patients in the Stable Cognitive Respiratory cluster, displaying good cognitive performance and mild respiratory symptoms, might benefit from preventive and maintenance strategies. Cognitive behavioral techniques and psychosocial support could foster effective self-management and adaptation to chronic health conditions.

These findings open new avenues for personalized treatment, emphasizing the potential of customizing treatment strategies to the specific dynamics of cognitive, affective, and respiratory interactions in enhancing clinical care.

## Figures and Tables

**Table 1:** Study Objectives.

Objective	Description	Purpose
Identification and Description of Patient Clusters	Analyzing and classifying patients into clusters based on cognitive performance, affective state, and severity of respiratory symptoms.	Deepening the understanding of how different patient profiles correlate with the progression of respiratory diseases and their management.
Assessment of Associations Between Cluster Characteristics	Determining statistical correlations between cognitive, affective, and respiratory disease severity variables within each cluster.	Identifying predictive factors that can influence disease progression and response to treatment, contributing to the personalization of therapeutic interventions.
Investigation of Clinical and Therapeutic Implications	Analyzing the impact of clustering results on medical practice and proposing changes to intervention strategies.	Improving integrated patient management, optimizing treatment and quality of life through personalized approaches.

**Table 2:** Results of normality tests for the evaluative measurement instruments used in the study.

Measurement Tool	Mean ± Standard Deviation	Statistic	*p* Value Shapiro–Wilk
MoCA	23.85 ± 4.434	0.8443	<0.001 *
MMSE	23.64 ± 4.291	0.8662	<0.001 *
HADS-D	8.39 ± 3.687	0.9222	<0.001 *
HADS-A	11.77 ± 6.174	0.9445	<0.001 *
CATS	25.54 ± 9.996	0.9448	0.0014 *
FEV1	54.13 ± 16.157	0.9643	0.0082 *

* *p* < 0.05—statistically significant.

**Table 3:** Comparison of cognitive assessment and mental health scores by MRC score.

Variable	Variable Type	MRC 1	MRC 2	MRC 3	MRC 4	Chi-Square	*p*-Value
MoCA	Normal	17 (30.91%)	14 (25.45%)	16 (29.09%)	8 (14.55%)	30.090	<0.001 *
	Mild	2 (6.06%)	5 (15.15%)	11 (33.33%)	15 (45.45%)		
	Moderate	0 (0.00%)	4 (40.00%)	6 (60.00%)	0 (0.00%)		
	Severe	0 (0.00%)	0 (0.00%)	0 (0.00%)	2 (100.00%)		
MMSE	Normal	17 (29.31%)	14 (24.14%)	15 (25.86%)	12 (20.69%)	25.299	0.003 *
	Mild	1 (3.57%)	8 (28.57%)	8 (28.57%)	11 (39.29%)		
	Moderate	1 (7.69%)	1 (7.69%)	10 (76.92%)	1 (7.69%)		
	Severe	0 (0.00%)	0 (0.00%)	0 (0.00%)	1 (100.00%)		
HADS-D	Normal	15 (42.86%)	13 (37.14%)	5 (14.29%)	2 (5.71%)	35.322	<0.001 *
	Mild	3 (6.25%)	7 (14.58%)	22 (45.83%)	16 (33.33%)		
	Moderate	1 (5.88%)	3 (17.65%)	6 (35.29%)	7 (41.18%)		
HADS-A	Normal	13 (50.00%)	10 (38.46%)	1 (3.85%)	2 (7.69%)	37.165	<0.0010 *
	Mild	3 (13.04%)	3 (13.04%)	11 (47.83%)	6 (26.09%)		
	Moderate	3 (7.69%)	7 (17.95%)	17 (43.59%)	12 (30.77%)		
	Severe	0 (0.00%)	3 (25.00%)	4 (33.33%)	5 (41.67%)		

* *p* < 0.05—statistically significant.

**Table 4:** Comparison of cognitive assessment and mental health scores by FEV1.

Variable	Severity	FEV1 Normal	FEV1 Mild	FEV1 Moderate	FEV1 Moderate Severe	FEV1 Severe	FEV1 Very Severe	Chi-Square	*p*-Value
MoCA	Normal	2 (3.64%)	13 (23.64%)	11 (20.00%)	8 (14.55%)	18 (32.73%)	3 (5.45%)	27.652	0.024 *
	Mild	4 (12.12%)	3 (9.09%)	1 (3.03%)	5 (15.15%)	14 (42.42%)	6 (18.18%)		
	Moderate	1 (10.00%)	1 (10.00%)	1 (10.00%)	5 (50.00%)	1 (10.00%)	2 (20.00%)		
	Severe	0 (0.00%)	0 (0.00%)	0 (0.00%)	0 (0.00%)	2 (100.00%)	0 (0.00%)		
MMSE	Normal	3 (5.17%)	13 (22.41%)	10 (17.24%)	8 (13.79%)	21 (36.21%)	3 (5.17%)	18.244	0.25
	Mild	3 (10.71%)	4 (14.29%)	2 (7.14%)	5 (17.86%)	8 (28.57%)	6 (21.43%)		
	Moderate	1 (7.69%)	0 (0.00%)	0 (0.00%)	5 (38.46%)	5 (38.46%)	2 (15.38%)		
	Severe	0 (0.00%)	0 (0.00%)	0 (0.00%)	0 (0.00%)	1 (100.00%)	0 (0.00%)		
HADS-D	Normal	1 (2.86%)	11 (31.43%)	7 (20.00%)	4 (11.43%)	10 (28.57%)	2 (5.71%)	17.561	0.063
	Mild	3 (6.25%)	5 (10.42%)	3 (6.25%)	10 (20.83%)	20 (41.67%)	7 (14.58%)		
	Moderate	3 (17.65%)	1 (5.88%)	2 (11.76%)	4 (23.53%)	5 (29.41%)	2 (11.76%)		
HADS-A	Normal	1 (3.85%)	8 (30.77%)	6 (23.08%)	2 (7.69%)	8 (30.77%)	2 (7.69%)	28.734	0.017 *
	Mild	3 (13.04%)	3 (13.04%)	3 (13.04%)	4 (17.39%)	8 (34.78%)	5 (21.74%)		
	Moderate	5 (12.82%)	5 (12.82%)	5 (12.82%)	11 (28.21%)	14 (35.90%)	4 (10.26%)		
	Severe	2 (16.67%)	1 (8.33%)	2 (16.67%)	1 (8.33%)	5 (41.67%)	5 (41.67%)		

* *p* < 0.05—statistically significant.

**Table 5:** Correlation between MRC scores and numerical evaluations of patients’ cognitive and affective performance.

Variable	MRC Group	Median (IQR)	Min–Max	Mean ± Standard Deviation	*p*-Value Kruskal–Wallis Test
MoCA	1	27.0 (1.0)	20–29	26.16 ± 2.14	0.040 *
	2	26.0 (7.0)	16–29	23.91 ± 4.54	
	3	25.0 (6.0)	13–29	23.36 ± 4.58	
	4	24.0 (5.0)	8–28	22.68 ± 4.98	
MMSE	1	26.0 (2.0)	19–29	25.63 ± 2.45	0.141
	2	26.0 (6.0)	13–29	24.13 ± 3.78	
	3	24.0 (7.0)	11–29	22.30 ± 5.19	
	4	23.0 (5.0)	9–28	23.44 ± 4.07	
HADS-D	1	5.0 (3.0)	3–14	5.68 ± 2.65	<0.001 *
	2	6.0 (3.5)	4–17	7.39 ± 3.43	
	3	9.0 (2.0)	2–16	9.24 ± 3.28	
	4	9.0 (4.0)	3–18	10.24 ± 3.79	
HADS-A	1	6.0 (3.0)	2–19	7.47 ± 4.49	<0.001 *
	2	10.0 (7.5)	2–24	10.22 ± 6.71	
	3	14.0 (7.0)	3–24	13.42 ± 5.27	
	4	13.0 (8.0)	4–24	14.28 ± 6.05	

* *p* < 0.05—statistically significant.

**Table 6:** Correlation between FEV1S scores and numerical evaluations of patients’ cognitive and affective performance.

Variable	FEV1S Group	Median (IQR)	Min–Max	Mean ± Standard Deviation	*p*-Value Kruskal–Wallis Test
MoCA	Normal	22.0 (5.0)	16–28	22.57 ± 4.31	0.034 *
	Mild	26.0 (1.0)	17–29	25.24 ± 3.68	
	Moderate	27.0 (2.0)	20–29	26.58 ± 2.27	
	Moderate severe	24.5 (9.5)	13–28	22.39 ± 5.23	
	Severe	26.0 (4.5)	8–28	23.51 ± 4.65	
	Very severe	24.0 (4.0)	16–28	23.00 ± 4.20	
MMSE	Normal	22.0 (5.0)	19–28	23.00 ± 3.37	0.043 *
	Mild	26.0 (2.0)	21–29	25.35 ± 2.47	
	Moderate	26.0 (0.5)	21–28	25.75 ± 1.86	
	Moderate severe	22.0 (6.75)	11–29	21.78 ± 5.17	
	Severe	26.0 (5.0)	9–29	23.89 ± 4.57	
	Very severe	22.0 (4.0)	13–27	21.36 ± 4.76	
HADS-D	Normal	8.0 (5.0)	4–14	9.71 ± 3.73	0.096
	Mild	5.0 (4.0)	2–17	6.47 ± 3.68	
	Moderate	6.5 (4.0)	4–15	7.58 ± 3.55	
	Moderate severe	9.0 (2.0)	4–16	9.33 ± 3.80	
	Severe	8.0 (3.5)	3–18	8.54 ± 3.56	
	Very severe	9.0 (1.5)	4–17	9.36 ± 3.47	
HADS-A	Normal	16.0 (6.0)	12–24	16.86 ± 4.85	0.033 *
	Mild	8.0 (8.0)	2–22	9.12 ± 5.95	
	Moderate	6.5 (5.75)	4–24	10.42 ± 7.89	
	Moderate severe	13.0 (7.5)	2–24	12.78 ± 5.57	
	Severe	12.0 (7.5)	3–24	12.34 ± 6.06	
	Very severe	9.0 (7.0)	3–19	10.64 ± 4.88	

* *p* < 0.05—statistically significant.

**Table 7:** Cluster analysis: patient profile identification based on cognitive, affective, and respiratory Characteristics.

Variable	Variable Type	Cluster 0 (*n* = 50) Mean ± Std. Deviation /Number and %	Cluster 1 (*n* = 12) Mean ± Std. Deviation /Number and %	Cluster 2 (*n* = 38) Mean ± Std. Deviation /Number and %
MoCA	Continue	24.56 ± 2.80	14.58 ± 3.02	25.84 ± 2.62
MMSE	Continue	24.00 ± 3.50	16.50 ± 4.62	25.42 ± 2.58
HADS-D	Continue	8.96 ± 2.82	12.33 ± 4.52	6.39 ± 3.19
HADS-A	Continue	13.08 ± 5.48	18.17 ± 4.30	8.03 ± 5.16
MRC	Categorical (1)	-	-	19 (50%)
	Categorical (2)	-	4 (33%)	2 (50%)
	Categorical (3)	27 (54%)	6 (50%)	-
	Categorical (4)	23 (46%)	2 (16.67%)	-
CAT	Continue	28.84 ± 8.42	24.25 ± 13.02	21.61 ± 9.58
FEV1	Continue	50.86 ± 13.98	53.42 ± 17.90	58.66 ± 17.56

Clustering performed using k-means clustering method with k = 3.

**Table 8:** The method of identifying the three clusters.

Variable	Cluster 0 (Moderate Cognitive Respiratory)	Cluster 1 (Severe Cognitive Respiratory)	Cluster 2 (Stable Cognitive Respiratory)
MoCA	≥20 and <26	<20	≥26
MMSE	≥21 and <25	<21	≥25
HADS-D	≥7 and ≤11	≥12	<7
HADS-A	≥9 and ≤16	≥17	<9
MRC	3 or 4	3 or 4, but especially 4	1 or 2
CAT	≥25 and ≤35	≥20 and ≤30	≤25
FEV1	≥40 and ≤60	≥50 and ≤65	≥55

## Data Availability

Data is contained within the article: The original contributions presented in the study are included in the article, further inquiries can be directed to the corresponding author/s.
